# Supplementary material for: Multiple imputation using auxiliary imputation variables that only predict missingness can increase bias due to data missing not at random
Source: BMC Med Res Methodol. 2024 Oct 7;24:231. doi: 10.1186/s12874-024-02353-9 (PMC11457445; doi:10.1186/s12874-024-02353-9)
Supplement: Supplementary file 1 — Supplementary Material 1 [file 12874_2024_2353_MOESM1_ESM.docx]

**Supplementary Material:** Multiple imputation using auxiliary imputation variables that only predict missingness can increase bias due to data missing not at random

Elinor Curnow^1,2^, Rosie P Cornish^1,2^, Jon E Heron^1,2^, James R Carpenter^3,4^, Kate Tilling^1,2^

^1^ Department of Population Health Sciences, Bristol Medical School, University of Bristol, Bristol, UK

^2^ Medical Research Council Integrative Epidemiology Unit at the University of Bristol, University of Bristol, Bristol, UK

^3^ Department of Medical Statistics, London School of Hygiene and Tropical Medicine, University of London, London, UK

^4^ Medical Research Council Clinical Trials Unit at University College London, University of London, London, UK

**Corresponding author:** Elinor Curnow, Population Health Sciences, Bristol Medical School, University of Bristol, Oakfield House, Oakfield Grove, Bristol BS8 2BN, UK

Telephone: +44 117 455 6622

Email: elinor.curnow@bristol.ac.uk, ORCID iD: 0000-0002-3109-3647

*Section S1. Scenario 1. Derivation of the theoretical expression for the maximum additional bias of the MI estimator when a continuous outcome Y is missing not at random, with missingness caused by Y itself, when the imputation model includes an auxiliary variable Z that predicts missingness but not the missing values*

As per the main text, we assume that the joint distribution of *Y*, *X*, *Z*, and *R*, *f*(*Y*, *X*, *Z*, *R*), is multivariate normal, with mean **µ**, covariance matrix **∑**, with associated univariate normal distributions defined as follows: *Y* = $\beta_{YX}$*X* + $\varepsilon_{Y}$ where $\varepsilon_{Y}$ ~ N(0, $\sigma_{Y}^{2}$); *X* ~ N($\mu_{X}$, $\sigma_{X}^{2}$);

*Z* ~ N($\mu_{Z}$, $\sigma_{Z}^{2}$); *R* = $\beta_{RY}$*Y* + $\beta_{RZ}$*Z* + $\varepsilon_{R}$ where $\varepsilon_{R}$ ~ N(0, $\sigma_{R}^{2}$)

Hence,

**µ** = $\left( \begin{aligned} \beta_{YX} \mu_{X} \\ \mu_{X} \\ \mu_{Z} \\ \beta_{RY} \beta_{YX} \mu_{X}+ \beta_{RZ} \mu_{Z} \end{aligned} \right)$

and

**∑** = $\left( \begin{matrix} \beta_{YX}^{2}\sigma_{X}^{2}+\sigma_{Y}^{2} & {\beta_{YX}\sigma}_{X}^{2} & 0 & \beta_{RY}(\beta_{YX}^{2}\sigma_{X}^{2}+\sigma_{Y}^{2}) \\ {\beta_{YX}\sigma}_{X}^{2} & \sigma_{X}^{2} & 0 & {\beta_{YX}\beta_{RY}\sigma}_{X}^{2} \\ 0 & 0 & \sigma_{Z}^{2} & {\beta_{RZ}\sigma}_{Z}^{2} \\ \beta_{RY}(\beta_{YX}^{2}\sigma_{X}^{2}+\sigma_{Y}^{2}) & {\beta_{YX}\beta_{RY}\sigma}_{X}^{2} & {\beta_{RZ}\sigma}_{Z}^{2} & Var(R) \end{matrix} \right)$

where $Var\left( R \right)$= $\beta_{RZ}^{2}\sigma_{Z}^{2}+\beta_{RY}^{2}(\beta_{YX}^{2}\sigma_{X}^{2}+\sigma_{Y}^{2})+\sigma_{R}^{2}$

We further assume that each of *Y* and *R* is a linear combination of the variables causing it plus an error term (with *X* and *Z* having no direct causes), with no interactions, all errors uncorrelated, no model mis-specification, and no measurement error, and that an ordinary least squares (OLS) estimator is used to obtain estimates in both analysis and imputation models.

**General expression for the magnitude of the MI estimator of** $\boldsymbol{\beta}_{\boldsymbol{YX}}$

We first derive a general expression for the magnitude of the MI estimator of the exposure regression coefficient, $\beta_{YX}$, following the argument of Curnow *et al.* (2023) (<https://doi.org/10.3389/fepid.2023.1237447>). As per the main text, we assume that MI is performed by replacing missing values of *Y* with draws from a linear regression model. We first consider the case in which the imputation model only includes *X* as a predictor. Then it follows, using the argument (and notation) from Chapter 2, Section 2.7.4, of Carpenter *et al.* (2023) (<https://doi.org/10.1002/9781119756118>), that the expected value of the MI estimator, $E(\hat{\beta}_{YX}^{MI})$, equals the expected value of the estimator of $\beta_{YX}$ based on records with observed values of *Y* (where this estimator is denoted by $\hat{\beta}_{YX}^{OBS}$), taking expectations first over the imputation distribution, given the set of observed values of *Y* (denoted by $Y_{OBS}$), and then over $Y_{OBS}$ itself. This reduces to: $E(\hat{\beta}_{YX}^{MI})$ = $E_{Y_{OBS}}\left( \hat{\beta}_{YX}^{OBS} \right)$.

The records with observed values of *Y* are those for which the missingness indicator, $\text{R}_{\text{ind}}$, equals 1, or equivalently, those for which *R* ≤ *r* (where, as per the main text, $\pi_{1}$ = P(*R_ind_* = 1) = P(*R* ≤ r) = $\Phi\left( \frac{r - \mu_{R}}{\sqrt{V_{R}}} \right)$, with $\Phi(.)$ denoting the cumulative distribution function of the standard normal distribution and $\mu_{R}$ and $V_{R}$ denoting the mean and variance of *R*, respectively). Hence,

$E(\hat{\beta}_{YX}^{MI})$ = $E_{Y_{OBS}}(\hat{\beta}_{YX|R_{ind}=1})$ = $E_{Y_{OBS}}(\hat{\beta}_{YX|R \leq r})$ =$\int_{-\infty}^{r} E_{Y_{OBS}}(\hat{\beta}_{YX|R=s}) p\left( s \right)ds$

where *p*(*s*) denotes the probability density at *R* = *s* given *s* $\in(-\infty, \text{r}]$, that is, *p*(*s*) = P(*R* = *s* | *R* ≤ *r*).

As per Curnow *et al.* (2023), the magnitude of $E(\hat{\beta}_{YX}^{MI})$ will vary according to the proportion of missing data, equalling $\beta_{YX}$ when there are no missing values, and approaching $\beta_{YX|R}$ as the proportion of missing values tends to one.

To gain an intuitive understanding of this result, we first consider the case where there are no missing data. We will assume, without loss of generality, that *R* has a standard normal distribution *i.e.* $\mu_{R}$ = 0 and $V_{R}$ = 1. If there are no missing values, $p(s)$ equals $\varphi\left( s \right)$ (where $\varphi\left( . \right)$ denotes the probability density function of the standard normal distribution) and $E(\hat{\beta}_{YX}^{MI})$ = $\int_{-\infty}^{\infty} E_{Y_{OBS}}(\hat{\beta}_{YX|R=s}) \varphi\left( s \right)ds$, which is the marginal coefficient, $\beta_{YX}$. Therefore, as expected, the MI estimator is unbiased if all records are complete. As the proportion of missing values tends to one (or equivalently, since r = $\Phi^{-1}\left( \pi_{1} \right)$, as *r* tends to $-\infty$), we use a heuristic argument to evaluate $E(\hat{\beta}_{YX}^{MI})$: consider a value of *r* such that the probability density for a value less than *r* is approximately equal to 0 *i.e.* $\varphi\left( s \right)$ ≈ 0 for all *s* < *r*. In this case, $p(s)$ ≈ 0 for all *s* < *r*, with $p(r)$ ≈ 1. Then, (using summation to approximate integration), $E(\hat{\beta}_{YX}^{MI})$ = $\lim_{r\to-\infty}\int_{-\infty}^{r} E_{Y_{OBS}}(\hat{\beta}_{YX|R=s}) p\left( s \right)ds$ ≈ $E_{Y_{OBS}}(\hat{\beta}_{YX|R=r}) p\left( r \right)$ $+\sum_{s<r} E_{Y_{OBS}}(\hat{\beta}_{YX|R=s}) p\left( s \right)$ = $\beta_{YX|R=r}$.

Similarly, when the imputation model includes *X* and *Z* as predictors, following the same argument as above and noting that $\beta_{YX|Z}$ is equivalent to $\beta_{YX}$ in our scenario because *X* is independent of *Z* by construction, the MI estimator is unbiased if all records are complete, and tends to $\beta_{YX|Z, R=r}$ as the proportion of missing values tends to one.

Thus, it follows that, in general, the additional bias of the MI estimator from including *Z*, as well as *X*, in the imputation model for *Y* is equal to: $\int_{-\infty}^{r} E_{Y_{OBS}}\left( \hat{\beta}_{YX|Z,R=s}- \hat{\beta}_{YX|R=s} \right) p(s)ds$, with maximum additional bias equal to $\beta_{YX|Z,R=r}- \beta_{YX|R=r}$.

**Derivation of maximum additional bias equation in terms of the direct effect sizes**

We can express $\beta_{YX|R=r}$ and $\beta_{YX|Z,R=r}$ in terms of the direct effect sizes and error variances using standard results for the joint conditional distribution of *Y*, *X*, and *Z,* given *R*:

*f*(*Y*, *X*, *Z* | *R* = *r*) is multivariate normal with covariance matrix **∑***, where

**∑*** = $\left( \begin{matrix} \beta_{YX}^{2}\sigma_{X}^{2}+\sigma_{Y}^{2} & {\beta_{YX}\sigma}_{X}^{2} & 0 \\ {\beta_{YX}\sigma}_{X}^{2} & \sigma_{X}^{2} & 0 \\ 0 & 0 & \sigma_{Z}^{2} \end{matrix} \right)- \frac{1}{Var(R)}\times\left( \begin{matrix} \beta_{RY}(\beta_{YX}^{2}\sigma_{X}^{2}+\sigma_{Y}^{2}) \\ {\beta_{YX}\beta_{RY}\sigma}_{X}^{2} \\ {\beta_{RZ}\sigma}_{Z}^{2} \end{matrix} \right)\left( \begin{matrix} \beta_{RY}(\beta_{YX}^{2}\sigma_{X}^{2}+\sigma_{Y}^{2}) & {\beta_{YX}\beta_{RY}\sigma}_{X}^{2} & \beta_{RZ}\sigma_{Z}^{2} \end{matrix} \right)$

= $\left( \begin{matrix} \beta_{YX}^{2}\sigma_{X}^{2}+\sigma_{Y}^{2} & {\beta_{YX}\sigma}_{X}^{2} & 0 \\ {\beta_{YX}\sigma}_{X}^{2} & \sigma_{X}^{2} & 0 \\ 0 & 0 & \sigma_{Z}^{2} \end{matrix} \right)- \frac{1}{Var(R)}\times\left( \begin{matrix} \beta_{RY}^{2}{(\beta_{YX}^{2}\sigma_{X}^{2}+\sigma_{Y}^{2})}^{2} & \beta_{YX}\beta_{RY}^{2}(\beta_{YX}^{2}\sigma_{X}^{2}+\sigma_{Y}^{2})\sigma_{X}^{2} & \beta_{RY}\beta_{RZ}(\beta_{YX}^{2}\sigma_{X}^{2}+\sigma_{Y}^{2})\sigma_{Z}^{2} \\ \beta_{YX}\beta_{RY}^{2}(\beta_{YX}^{2}\sigma_{X}^{2}+\sigma_{Y}^{2})\sigma_{X}^{2} & \beta_{YX}^{2}\beta_{RY}^{2}\sigma_{X}^{4} & \beta_{YX}{\beta_{RY}\beta_{RZ}\sigma}_{X}^{2}\sigma_{Z}^{2} \\ \beta_{RY}\beta_{RZ}(\beta_{YX}^{2}\sigma_{X}^{2}+\sigma_{Y}^{2})\sigma_{Z}^{2} & \beta_{YX}{\beta_{RY}\beta_{RZ}\sigma}_{X}^{2}\sigma_{Z}^{2} & \beta_{RZ}^{2}\sigma_{Z}^{4} \end{matrix} \right)$

Hence, $\beta_{YX|R=r}$ = $\frac{Cov(X,Y|R=r)}{Var(X|R=r)}$ = $\frac{\beta_{YX}\sigma_{X}^{2}\{Var\left( R \right) - \beta_{RY}^{2}\left( \beta_{YX}^{2}\sigma_{X}^{2}+\sigma_{Y}^{2} \right)\}}{\sigma_{X}^{2}\{Var\left( R \right) - \beta_{YX}^{2}\beta_{RY}^{2}\sigma_{X}^{2}\}}$

= $\frac{\beta_{YX}\{\beta_{RZ}^{2}\sigma_{Z}^{2}+\beta_{YX}^{2}\beta_{RY}^{2}\sigma_{X}^{2}+\beta_{RY}^{2}\sigma_{Y}^{2}+\sigma_{R}^{2} - \beta_{YX}^{2}\beta_{RY}^{2}\sigma_{X}^{2} - \beta_{RY}^{2}\sigma_{Y}^{2}\}}{\beta_{RZ}^{2}\sigma_{Z}^{2}+\beta_{YX}^{2}\beta_{RY}^{2}\sigma_{X}^{2}+\beta_{RY}^{2}\sigma_{Y}^{2}+\sigma_{R}^{2} - \beta_{YX}^{2}\beta_{RY}^{2}\sigma_{X}^{2}}$

= $\frac{\beta_{YX}\{\beta_{RZ}^{2}\sigma_{Z}^{2} + \sigma_{R}^{2}\}}{\beta_{RZ}^{2}\sigma_{Z}^{2}+\beta_{RY}^{2}\sigma_{Y}^{2}+\sigma_{R}^{2}}$

= $\beta_{YX} \times\left\{ 1-\frac{\beta_{RY}^{2}\sigma_{Y}^{2}}{\beta_{RY}^{2}\sigma_{Y}^{2}+\beta_{RZ}^{2}\sigma_{Z}^{2}+\sigma_{R}^{2}} \right\}$ as per Equation 2.1 in the main text.

Using a similar approach, the joint conditional distribution of *Y* and *X* given *Z* and *R*, *f*(*Y*, *X* | *Z*=z, *R*=r), is multivariate normal with covariance matrix **∑****, where

**∑**** = $\left( \begin{matrix} \beta_{YX}^{2}\sigma_{X}^{2}+\sigma_{Y}^{2} & {\beta_{YX}\sigma}_{X}^{2} \\ {\beta_{YX}\sigma}_{X}^{2} & \sigma_{X}^{2} \end{matrix} \right)- \frac{1}{\sigma_{Z}^{2}Var\left( R \right)-{\beta_{RZ}^{2}\sigma}_{Z}^{4}}\times$

$$\left( \begin{matrix} 0 & \beta_{RY}(\beta_{YX}^{2}\sigma_{X}^{2}+\sigma_{Y}^{2}) \\ 0 & {\beta_{YX}\beta_{RY}\sigma}_{X}^{2} \end{matrix} \right)\left( \begin{matrix} Var(R) & {-\beta}_{RZ}\sigma_{Z}^{2} \\ {{-\beta}_{RZ}\sigma}_{Z}^{2} & \sigma_{Z}^{2} \end{matrix} \right)\left( \begin{matrix} 0 & 0 \\ \beta_{RY}(\beta_{YX}^{2}\sigma_{X}^{2}+\sigma_{Y}^{2}) & {\beta_{YX}\beta_{RY}\sigma}_{X}^{2} \end{matrix} \right)$$

= $\left( \begin{matrix} \beta_{YX}^{2}\sigma_{X}^{2}+\sigma_{Y}^{2} & {\beta_{YX}\sigma}_{X}^{2} \\ {\beta_{YX}\sigma}_{X}^{2} & \sigma_{X}^{2} \end{matrix} \right)- \frac{1}{\sigma_{Z}^{2}Var\left( R \right)-{\beta_{RZ}^{2}\sigma}_{Z}^{4}}\times$

$$\left( \begin{matrix} 0 & \beta_{RY}(\beta_{YX}^{2}\sigma_{X}^{2}+\sigma_{Y}^{2}) \\ 0 & {\beta_{YX}\beta_{RY}\sigma}_{X}^{2} \end{matrix} \right)\left( \begin{matrix} {-\beta}_{RZ}\beta_{RY}(\beta_{YX}^{2}\sigma_{X}^{2}+\sigma_{Y}^{2})\sigma_{Z}^{2} & -\beta_{YX}\beta_{RZ}\beta_{RY}\sigma_{X}^{2}\sigma_{Z}^{2} \\ \beta_{RY}(\beta_{YX}^{2}\sigma_{X}^{2}+\sigma_{Y}^{2})\sigma_{Z}^{2} & \beta_{YX}\beta_{RY}\sigma_{X}^{2}\sigma_{Z}^{2} \end{matrix} \right)$$

= $\left( \begin{matrix} \beta_{YX}^{2}\sigma_{X}^{2}+\sigma_{Y}^{2} & {\beta_{YX}\sigma}_{X}^{2} \\ {\beta_{YX}\sigma}_{X}^{2} & \sigma_{X}^{2} \end{matrix} \right)- \frac{1}{\sigma_{Z}^{2}Var\left( R \right)-{\beta_{RZ}^{2}\sigma}_{Z}^{4}}\times\left( \begin{matrix} \beta_{RY}^{2}{(\beta_{YX}^{2}\sigma_{X}^{2}+\sigma_{Y}^{2})}^{2}\sigma_{Z}^{2} & \beta_{YX}\beta_{RY}^{2}(\beta_{YX}^{2}\sigma_{X}^{2}+\sigma_{Y}^{2})\sigma_{X}^{2}\sigma_{Z}^{2} \\ \beta_{YX}\beta_{RY}^{2}(\beta_{YX}^{2}\sigma_{X}^{2}+\sigma_{Y}^{2})\sigma_{X}^{2}\sigma_{Z}^{2} & \beta_{YX}^{2}\beta_{RY}^{2}\sigma_{X}^{4}\sigma_{Z}^{2} \end{matrix} \right)$

Hence, $\beta_{YX|Z=z,R=r}$ = $\frac{Cov(X,Y|Z=z,R=r)}{Var(X|Z=z,R=r)}$ = $\frac{\beta_{YX}\sigma_{X}^{2}\sigma_{Z}^{2}\left\{ Var\left( R \right) - {\beta_{RZ}^{2}\sigma}_{Z}^{2} - \beta_{RY}^{2}(\beta_{YX}^{2}\sigma_{X}^{2}+\sigma_{Y}^{2}) \right\}}{\sigma_{X}^{2}\sigma_{Z}^{2}\{Var\left( R \right) - {\beta_{RZ}^{2}\sigma}_{Z}^{2} - \beta_{YX}^{2}\beta_{RY}^{2}\sigma_{X}^{2}\}}$

= $\beta_{YX}\times\frac{\beta_{RZ}^{2}\sigma_{Z}^{2}+\beta_{RY}^{2}\left( \beta_{YX}^{2}\sigma_{X}^{2}+\sigma_{Y}^{2} \right)+\sigma_{R}^{2} - {\beta_{RZ}^{2}\sigma}_{Z}^{2} - \beta_{RY}^{2}\left( \beta_{YX}^{2}\sigma_{X}^{2}+\sigma_{Y}^{2} \right)}{\beta_{RZ}^{2}\sigma_{Z}^{2}+\beta_{RY}^{2}(\beta_{YX}^{2}\sigma_{X}^{2}+\sigma_{Y}^{2})+\sigma_{R}^{2} - {\beta_{RZ}^{2}\sigma}_{Z}^{2} - \beta_{YX}^{2}\beta_{RY}^{2}\sigma_{X}^{2}}$

= $\beta_{YX}\times\frac{\sigma_{R}^{2}}{\beta_{RY}^{2}\sigma_{Y}^{2}+\sigma_{R}^{2}}$ = $\beta_{YX}\times\left\{ 1-\frac{\beta_{RY}^{2}\sigma_{Y}^{2}}{\beta_{RY}^{2}\sigma_{Y}^{2}+\sigma_{R}^{2}} \right\}$ as per Equation 2.2 in the main text.

Note that $\beta_{YX|R=r}$ and $\beta_{YX|Z=z,R=r}$ do not depend on the specific values of *r* and *z*, hence we use the more general forms $\beta_{YX|R}$ and $\beta_{YX|Z,R}$ hereafter.

Recall that $\beta_{YX|R}$ = $\beta_{YX} \times\left\{ 1-\frac{\beta_{RY}^{2}\sigma_{Y}^{2}}{\beta_{RY}^{2}\sigma_{Y}^{2}+\sigma_{R}^{2}+\beta_{RZ}^{2}\sigma_{Z}^{2}} \right\}$. Thus, the maximum bias of the MI estimator due to *Y* being MNAR (using only *X* as a predictor in the imputation model for *Y*) is $-\frac{\beta_{YX}\beta_{RY}^{2}\sigma_{Y}^{2}}{\beta_{RY}^{2}\sigma_{Y}^{2}+\sigma_{R}^{2}+\beta_{RZ}^{2}\sigma_{Z}^{2}}$.

The maximum additional bias of the MI estimator (*i.e.* in addition to the bias due to *Y* being MNAR) from including *Z* as a predictor in the imputation model is $\beta_{YX}\beta_{RY}^{2}\sigma_{Y}^{2}\times\left\{ \frac{1}{\beta_{RY}^{2}\sigma_{Y}^{2}+\sigma_{R}^{2}+\beta_{RZ}^{2}\sigma_{Z}^{2}}-\frac{1}{\beta_{RY}^{2}\sigma_{Y}^{2}+\sigma_{R}^{2}} \right\}$ = $\frac{-\beta_{YX}\beta_{RY}^{2}\beta_{RZ}^{2}\sigma_{Y}^{2}\sigma_{Z}^{2}}{\left( \beta_{RY}^{2}\sigma_{Y}^{2}+\sigma_{R}^{2}+\beta_{RZ}^{2}\sigma_{Z}^{2} \right)\left( \beta_{RY}^{2}\sigma_{Y}^{2}+\sigma_{R}^{2} \right)}$ as per Equation 2.3 in the main text.

Or in other words, if bias amplification is defined as the bias of $\beta_{YX|Z,R}$ divided by the bias of $\beta_{YX|R}$, then maximum bias amplification = $\frac{-\beta_{YX}\beta_{RY}^{2}\sigma_{Y}^{2}}{\beta_{RY}^{2}\sigma_{Y}^{2}+\sigma_{R}^{2}}/\frac{-\beta_{YX}\beta_{RY}^{2}\sigma_{Y}^{2}}{\beta_{RY}^{2}\sigma_{Y}^{2}+\sigma_{R}^{2}+\beta_{RZ}^{2}\sigma_{Z}^{2}}$ = $\frac{\beta_{RY}^{2}\sigma_{Y}^{2}+\sigma_{R}^{2}+\beta_{RZ}^{2}\sigma_{Z}^{2}}{\beta_{RY}^{2}\sigma_{Y}^{2}+\sigma_{R}^{2}}$ = $1+\frac{\beta_{RZ}^{2}\sigma_{Z}^{2}}{\beta_{RY}^{2}\sigma_{Y}^{2}+\sigma_{R}^{2}}$, that is, the maximum bias due to *Y* being MNAR is amplified by a factor of $\left\{ 1+\frac{\beta_{RZ}^{2}\sigma_{Z}^{2}}{\beta_{RY}^{2}\sigma_{Y}^{2}+\sigma_{R}^{2}} \right\}$ when *Z* is included in the imputation model for *Y*, as per Equation 2.4 in the main text.

*Section S2. Scenario 1. Verification of the theoretical expression for the maximum additional bias of the MI estimator when a continuous outcome Y is missing not at random, with missingness caused by Y itself, when the imputation model includes an auxiliary variable Z that predicts missingness but not the missing values*

The theoretical expressions for the maximum bias of the MI estimator due to Y being MNAR (when the imputation model includes only *X*), as well as the maximum additional bias of the MI estimator when the imputation model includes *X* and *Z*, were verified using simulation. We used 1000 simulations, and each simulated dataset contained 100,000 observations. In each simulated dataset, the values of each coefficient ($\beta_{YX}$, $\beta_{RZ}$, *etc*.) and each error variance ($\sigma_{X}^{2}$, $\sigma_{Z}^{2}$, *etc.*) were sampled from a uniform distribution *U*(0, 2). For simplicity, $\mu_{X}$ and $\mu_{Z}$ were set equal to zero (note that the equations do not depend on these parameters). Data were then generated using the specified models for *Y*, *X*, *Z*, and *R*, as per Section S1, above. The two bias quantities were calculated using the theoretical expressions (Equations 2.1 and 2.3 in the main text – note that verification of these two equations also implicitly verifies Equations 2.2 and 2.4). They were also estimated empirically by calculating the difference in the *X* coefficient when fitting a linear regression of (i) *Y* on *X*, (ii) *Y* on *X*, conditional on *R*, and (iii) *Y* on *X*, conditional on *R* and *Z* (with the difference between the coefficient from models (i) and (ii) used to estimate the maximum bias, and the difference between the coefficient from models (ii) and (iii) used to estimate the maximum additional bias).

The median difference between the theoretical and empirical values of maximum bias and maximum additional bias was 0.000 (5^th^ – 95^th^ percentile: -0.007 - 0.009) and 0.000 (5^th^ – 95^th^ percentile: -0.004 - 0.005), respectively. Therefore, we conclude that the theoretical expressions are correct.

*Section S3. Description of simulation studies to assess the additional bias of the MI estimator from including a predictor of missingness but not the missing values in the imputation model when a binary outcome Y, or continuous or binary exposure X, is partially observed.*

We performed simulation studies to assess the additional bias of the MI estimator from including a predictor of missingness but not the missing values in the imputation model when (i) a binary outcome *Y*, (ii) a continuous exposure *X*, or (iii) a binary exposure *X* were partially observed, in each of the three scenarios discussed in the main paper, considering settings in which we would expect the MI estimator to be biased. For example, in Scenario 1, we considered the setting in which a binary outcome *Y* was partially observed, but not settings in which a continuous or binary exposure *X* was partially observed (because *X* was MAR in Scenario 1 and hence MI using a correctly specified imputation model would be valid).

In each setting, 1000 simulated datasets of size 1000 were generated using the data generating mechanisms described below. We used moderate values of the direct effect sizes (relative to the error variances, which were all equal to one), with all direct effect sizes set to 0.00, 0.50, or 1.00 and the mean of each variable equal to zero. We then set 50% of values of the partially observed variable to missing (in each case, by setting values to missing if *R* > 0). Additional bias was calculated as the average of the per-simulation estimates (where the per-simulation estimate was calculated as the difference between the MI estimate using *Z* and the other analysis model variable as predictors, and the MI estimate using just the other analysis model variable as a predictor in the imputation model, with five imputations in each setting).

*Data generating mechanisms*

In setting (i) (binary outcome *Y* is partially observed), all variables except *Y* were related as defined in Sections S1, S4, and S5 (for Scenarios 1, 2, and 3, respectively), with *Y* defined as *logit*{P(*Y*=1)} *=* $\beta_{YX}$*X* in Scenarios 1 and 3, and *logit*{P(*Y*=1)} *=* $\beta_{YX}$*X* + $\beta_{YU}$*U* in Scenario 2. In setting (ii) (continuous exposure *X* is partially observed), all variables were related as defined in Sections S4 and S5 (for Scenarios 2 and 3, respectively). In setting (iii) (binary exposure *X* is partially observed), all variables except *X* were related as defined in Sections S4 and S5 (for Scenarios 2 and 3, respectively), with *X* defined as binary, with probability 0.5 of a value of 0 or 1.

*Results*

Additional bias (and also total bias in Scenario 2) of the MI estimate of $\beta_{YX}$ when the imputation model includes a predictor of missingness, *Z*, when 50% of values are missing for either a binary outcome *Y* (Figures S1, S2-3, and S5 for Scenarios 1-3, respectively), or a binary exposure *X* (Figures S4 and S6 for Scenarios 2 and 3, respectively) are shown below (with results for a continuous exposure *X* illustrated in Figures 4 and 7 in the main text). Note that in these plots, additional and total bias do not take their maximum values because the proportion of missing data is 50% (rather than tending to 100%). However, as per Curnow *et al.* (2023) (<https://doi.org/10.3389/fepid.2023.1237447>), the maximum values are likely to be approximately double the magnitude depicted in these plots.

*Figure S1. Scenario 1. Additional bias of the MI estimate of* $\beta_{YX}$ *when binary outcome Y is missing not at random, with missingness caused by Y itself, when the imputation model includes an auxiliary variable Z that predicts missingness but not the missing values. Results shown when 50% of values are missing, varying the direct effect sizes* $\beta_{YX}$, $\beta_{RY}$, and $\beta_{RZ}$*. The distribution of additional bias in each box-plot is due to variation in* $\beta_{RY}$*.*

*
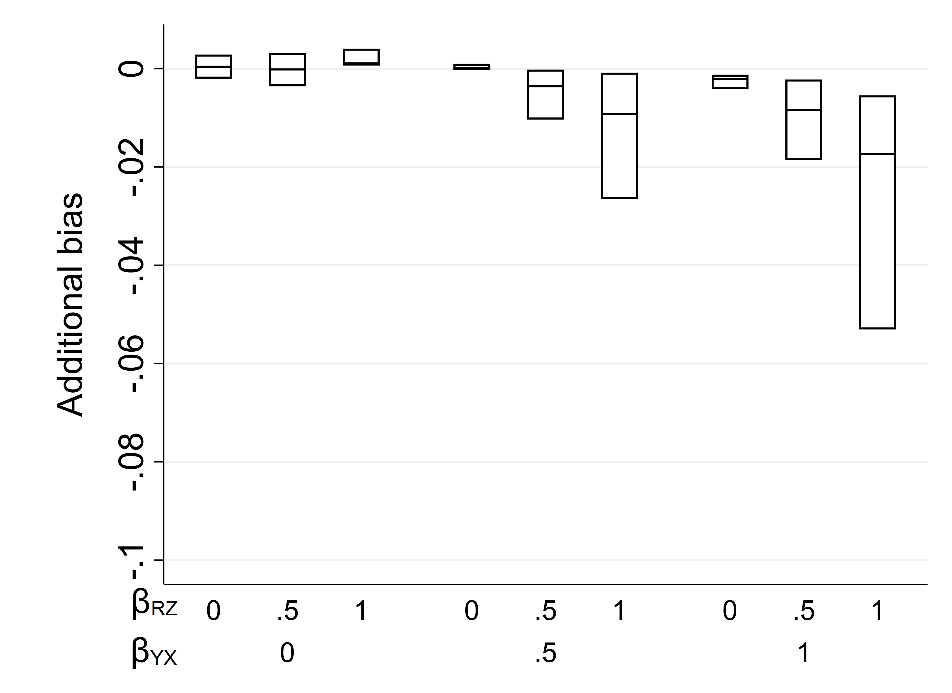
*

*Figure S2. Scenario 2. Additional bias of the MI estimate of* $\beta_{YX}$ *when binary outcome Y is missing not at random, with missingness related to Y via an unmeasured variable U, when the imputation model includes an auxiliary variable Z that predicts missingness but not the missing values. Results shown when 50% of values are missing, varying the direct effect sizes* $\beta_{YX}$, $\beta_{YU}$, $\beta_{RU}$, and $\beta_{RZ}$*. The distribution of additional bias in each box-plot is averaged over the values of* $\beta_{YU}$ *and* $\beta_{RU}$*.*

*
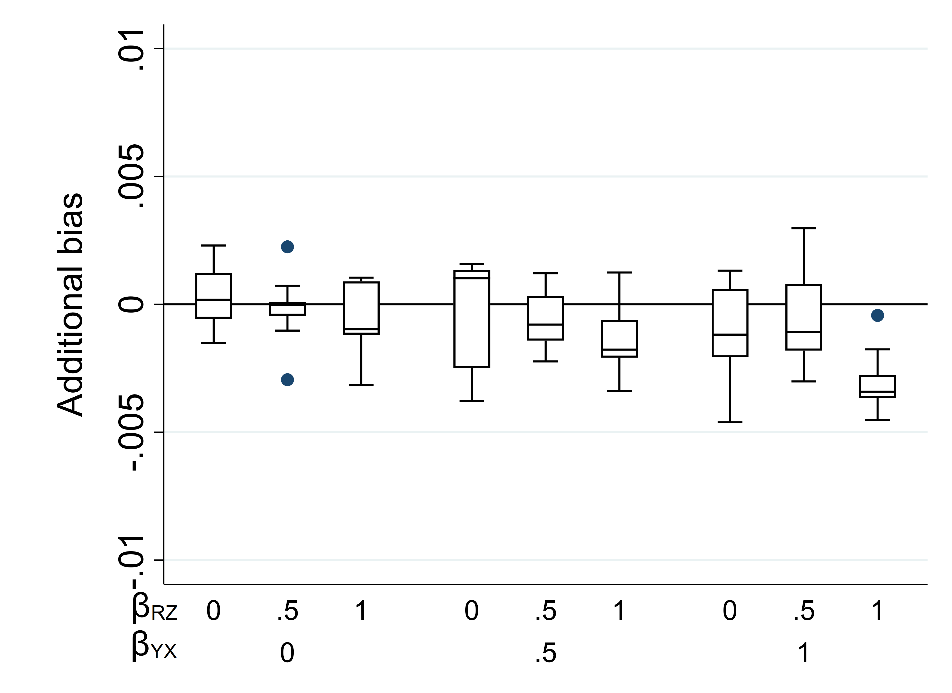
*

*Figure S3. Scenario 2. Total bias of the MI estimate of* $\beta_{YX}$ *when binary outcome Y is missing not at random, with missingness related to Y via an unmeasured variable U, when the imputation model includes an auxiliary variable Z that predicts missingness but not the missing values. Results shown when 50% of values are missing, varying the direct effect sizes* $\beta_{YX}$, $\beta_{YU}$, $\beta_{RU}$, and $\beta_{RZ}$*. The distribution of additional bias in each box-plot is averaged over the values of* $\beta_{YU}$ *and* $\beta_{RU}$*.*

*
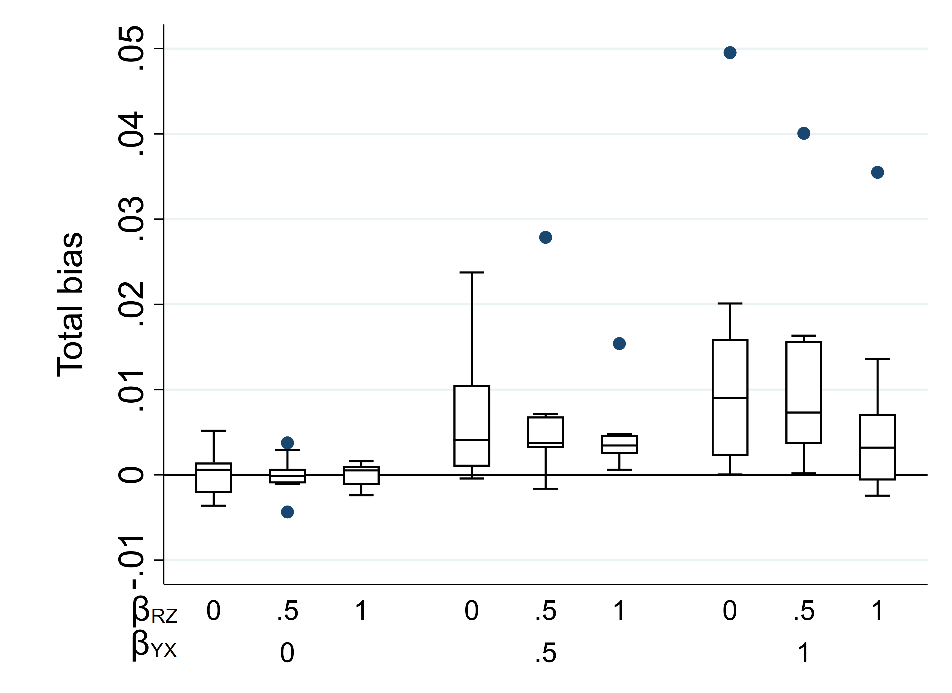
*

*Figure S4. Scenario 2. Additional bias of the MI estimate of* $\beta_{YX}$ *when binary exposure X is missing not at random, with missingness related to Y via an unmeasured variable U, when the imputation model includes an auxiliary variable Z that predicts missingness but not the missing values. Results shown when 50% of values are missing, varying the direct effect sizes* $\beta_{YX}$, $\beta_{YU}$, $\beta_{RU}$, and $\beta_{RZ}$*. The distribution of additional bias in each box-plot is averaged over the values of* $\beta_{YU}$ *and* $\beta_{RU}$*.*

*
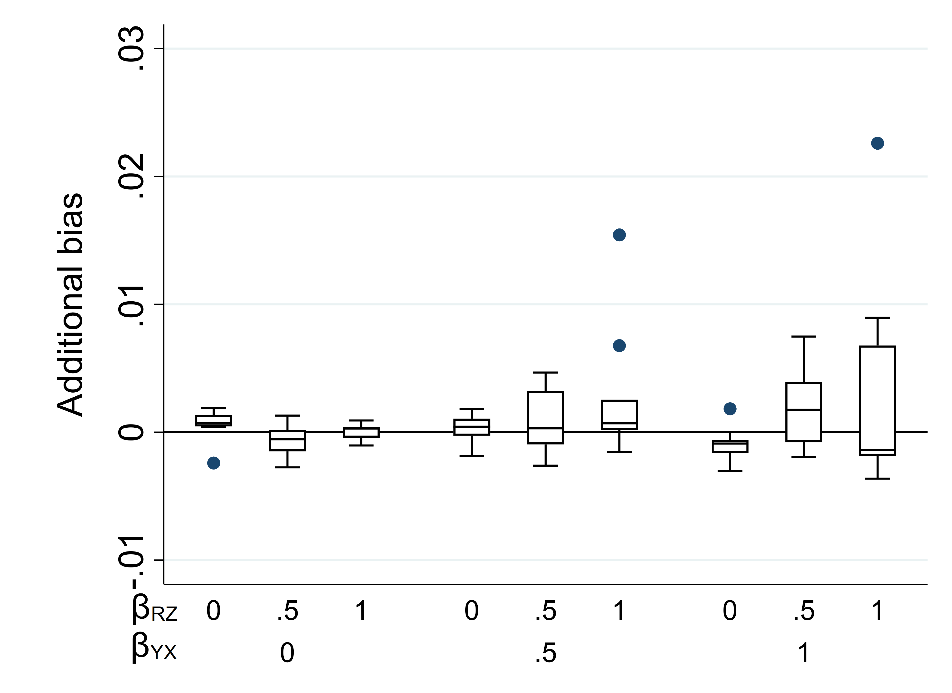
*

*Figure S5. Scenario 3. Additional bias of the MI estimate of* $\beta_{YX}$ *when binary outcome Y is missing not at random, with missingness caused by Y and X, when the imputation model includes an auxiliary variable Z that predicts missingness but not the missing values. Results shown when 50% of values are missing, varying the direct effect sizes* $\beta_{YX}$, $\beta_{RY}$, $\beta_{RX}$, and $\beta_{RZ}$*. The distribution of additional bias in each box-plot is averaged over the values of* $\beta_{RY}$ *and* $\beta_{RX}$*.*

*
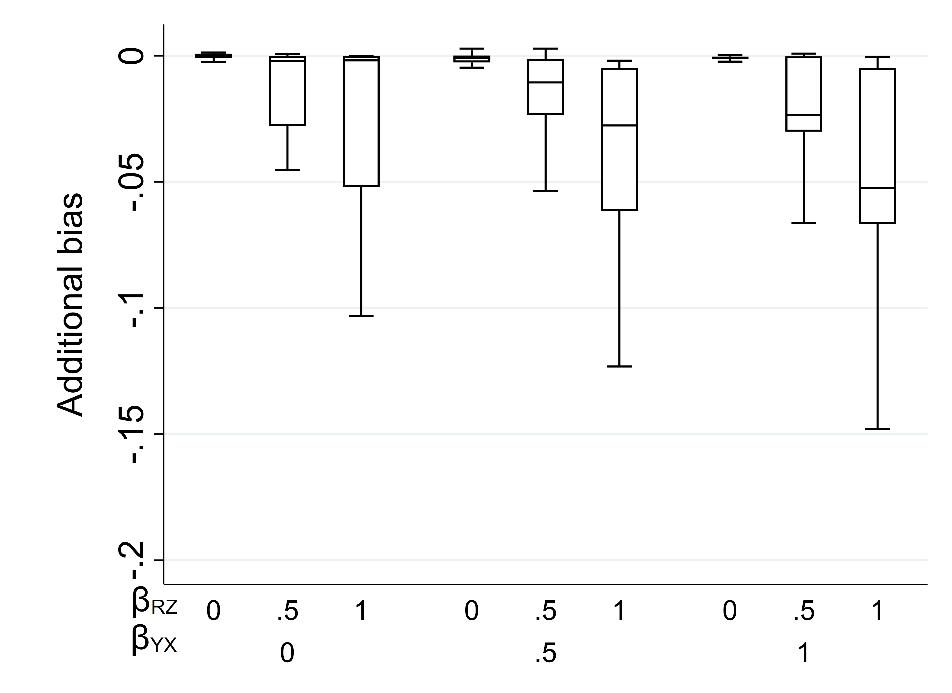
*

*Figure S6. Scenario 3. Additional bias of the MI estimate of* $\beta_{YX}$ *when binary exposure X is missing not at random, with missingness caused by Y and X, when the imputation model includes an auxiliary variable Z that predicts missingness but not the missing values. Results shown when 50% of values are missing, varying the direct effect sizes* $\beta_{YX}$, $\beta_{RY}$, $\beta_{RX}$, and $\beta_{RZ}$*. The distribution of additional bias in each box-plot is averaged over the values of* $\beta_{RY}$

*and* $\beta_{RX}$*.*


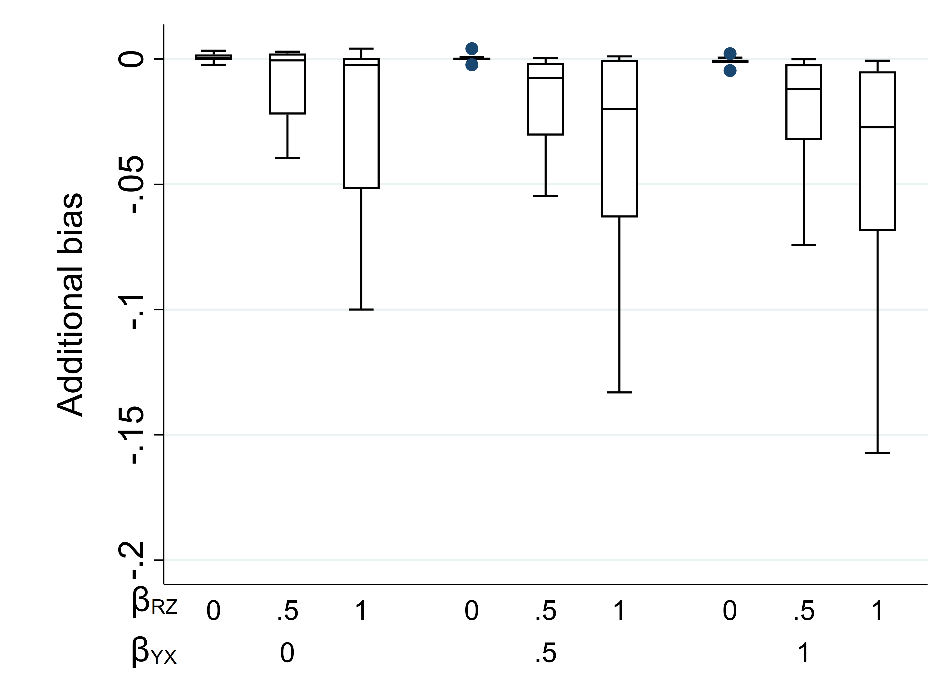


*Section S4. Scenario 2. Derivation of theoretical expressions for the maximum additional bias of (i) the MI estimator when a continuous outcome Y is partially observed and (ii) the Y coefficient in the imputation model for continuous exposure X when X is partially observed, when the imputation model includes an auxiliary variable Z that predicts missingness but not the missing values, given missingness is related to Y via an unmeasured variable U.*

As per the main text, in Scenario 2 we assume that the joint distribution of *Y*, *X*, *Z*, *U*, and *R*, *f*(*Y*, *X*, *Z*, U, R), is multivariate normal, with mean **µ**, covariance matrix **∑**, with associated univariate normal distributions defined as follows:

*Y* = $\beta_{YX}$*X* + $\beta_{YU}$*U* + $\varepsilon_{Y}$, where $\varepsilon_{Y}$ ~ N(0, $\sigma_{Y}^{2}$); *X* ~ N($\mu_{X}$, $\sigma_{X}^{2}$); *Z* ~ N($\mu_{Z}$, $\sigma_{Z}^{2}$); *U* ~ N($\mu_{U}$, $\sigma_{U}^{2}$);

*R* = $\beta_{RZ}$*Z* + $\beta_{RU}$*U* + $\varepsilon_{R}$, where $\varepsilon_{R}$ ~ N(0, $\sigma_{R}^{2}$).

We once again assume that each of *Y* and *R* is a linear combination of the variables causing it plus an error term (with *X*, *Z*, and *U* having no direct causes), with no interactions, all errors uncorrelated, no model mis-specification, and no measurement error, and that an ordinary least squares (OLS) estimator is used to obtain estimates in both analysis and imputation models.

Hence,

**µ** = $\left( \begin{aligned} \beta_{YX} \mu_{X}+ \beta_{YU} \mu_{U} \\ \mu_{X} \\ \mu_{Z} \\ \mu_{U} \\ \beta_{RZ} \mu_{Z}+\beta_{RU} \mu_{U} \end{aligned} \right)$

and

**∑** = $\left( \begin{matrix} \beta_{YX}^{2}\sigma_{X}^{2}+{\beta_{YU}^{2}\sigma}_{U}^{2}+\sigma_{Y}^{2} & {\beta_{YX}\sigma}_{X}^{2} & 0 & {\beta_{YU}\sigma}_{U}^{2} & {\beta_{YU}\beta_{RU}\sigma}_{U}^{2} \\ {\beta_{YX}\sigma}_{X}^{2} & \sigma_{X}^{2} & 0 & 0 & 0 \\ 0 & 0 & \sigma_{Z}^{2} & 0 & {\beta_{RZ}\sigma}_{Z}^{2} \\ {\beta_{YU}\sigma}_{U}^{2} & 0 & 0 & \sigma_{U}^{2} & {\beta_{RU}\sigma}_{U}^{2} \\ {\beta_{YU}\beta_{RU}\sigma}_{U}^{2} & 0 & {\beta_{RZ}\sigma}_{Z}^{2} & {\beta_{RU}\sigma}_{U}^{2} & Var(R) \end{matrix} \right)$

where $Var(R)$ = $\beta_{RZ}^{2}\sigma_{Z}^{2}+\beta_{RU}^{2}\sigma_{U}^{2}+\sigma_{R}^{2}$

**General expression for the magnitude of the maximum additional bias of (i) the MI estimator of** $\boldsymbol{\beta}_{\boldsymbol{YX}}$ **and (ii) the estimator of the *Y* coefficient from the imputation model for *X***

When *Y* is partially observed, we use the general expression for the maximum additional bias of the MI estimator from Section S1, namely, maximum additional bias equals $\beta_{YX|Z,R}-\beta_{YX|R}$.

When *X* is partially observed (again assuming that MI is performed by replacing missing values of *X* with draws from a linear regression model), the MI estimator of $\beta_{YX}$ will be unbiased only if the estimator of each imputation model coefficient, based on records with observed values of *X*, is unbiased. Briefly, using a similar argument to that in Section S1 and taking the *Y* coefficient from the imputation model for *X* as an example (denoting the true value of the *Y* coefficient by $\beta_{XY}$and its estimator based on records with observed values of *X* by $\hat{\beta}_{XY}^{OBS}$), we find:

1. When the imputation model includes only *Y* as a predictor, the magnitude of $\hat{\beta}_{XY}^{OBS}$ will vary according to the proportion of missing data, equalling $\beta_{XY}$ when there are no missing values, and approaching $\beta_{XY|R=r}$ as the proportion of missing values tends to one.
2. When the imputation model includes *Y* and *Z* as predictors (noting that $\beta_{XY|Z}$ is equivalent to $\beta_{XY}$ in our scenario because *Y* is independent of *Z* by construction), the magnitude of $\hat{\beta}_{XY}^{OBS}$ will again vary according to the proportion of missing data, equalling $\beta_{XY}$ when there are no missing values, and approaching $\beta_{XY|Z=z, R=r}$ as the proportion of missing values tends to one.

Therefore, the maximum additional bias of the *Y* coefficient when using *Y* and *Z* as predictors in the imputation model for *X* compared with just *Y* will be $\beta_{XY|Z=z, R=r}-\beta_{XY|R=r}$

**Derivation of maximum additional bias equations in terms of the direct effect sizes**

We can express $\beta_{YX|R=r}$, $\beta_{XY|R=r}$, $\beta_{XY|Z=z,R=r}$, and $\beta_{XY|Z=z,R=r}$ in terms of the direct effect sizes and error variances using standard results.

The joint conditional distribution of *Y*, *X*, *Z*, and *U* given *R*, *f*(*Y*, *X*, *Z*, *U* | *R*=r), is multivariate normal with covariance matrix **∑***, where:

**∑*** = $\left( \begin{matrix} \beta_{YX}^{2}\sigma_{X}^{2}+{\beta_{YU}^{2}\sigma}_{U}^{2}+\sigma_{Y}^{2} & {\beta_{YX}\sigma}_{X}^{2} & 0 & {\beta_{YU}\sigma}_{U}^{2} \\ {\beta_{YX}\sigma}_{X}^{2} & \sigma_{X}^{2} & 0 & 0 \\ 0 & 0 & \sigma_{Z}^{2} & 0 \\ {\beta_{YU}\sigma}_{U}^{2} & 0 & 0 & \sigma_{U}^{2} \end{matrix} \right)- \frac{1}{Var(R)}\times\left( \begin{matrix} {\beta_{YU}\beta_{RU}\sigma}_{U}^{2} \\ 0 \\ {\beta_{RZ}\sigma}_{Z}^{2} \\ {\beta_{RU}\sigma}_{U}^{2} \end{matrix} \right)\left( \begin{matrix} {\beta_{YU}\beta_{RU}\sigma}_{U}^{2} & 0 & {\beta_{RZ}\sigma}_{Z}^{2} & {\beta_{RU}\sigma}_{U}^{2} \end{matrix} \right)$

= $\left( \begin{matrix} \beta_{YX}^{2}\sigma_{X}^{2}+{\beta_{YU}^{2}\sigma}_{U}^{2}+\sigma_{Y}^{2} & {\beta_{YX}\sigma}_{X}^{2} & 0 & {\beta_{YU}\sigma}_{U}^{2} \\ {\beta_{YX}\sigma}_{X}^{2} & \sigma_{X}^{2} & 0 & 0 \\ 0 & 0 & \sigma_{Z}^{2} & 0 \\ {\beta_{YU}\sigma}_{U}^{2} & 0 & 0 & \sigma_{U}^{2} \end{matrix} \right)- \frac{1}{Var(R)}\times\left( \begin{matrix} \beta_{YU}^{2}\beta_{RU}^{2}\sigma_{U}^{4} & 0 & \beta_{YU}\beta_{RU}\beta_{RZ}\sigma_{U}^{2}\sigma_{Z}^{2} & \beta_{YU}\beta_{RU}^{2}\sigma_{U}^{4} \\ 0 & 0 & 0 & 0 \\ \beta_{YU}\beta_{RU}\beta_{RZ}\sigma_{U}^{2}\sigma_{Z}^{2} & 0 & \beta_{RZ}^{2}\sigma_{Z}^{4} & {\beta_{RU}\beta_{RZ}\sigma}_{U}^{2}\sigma_{Z}^{2} \\ \beta_{YU}\beta_{RU}^{2}\sigma_{U}^{4} & 0 & {\beta_{RU}\beta_{RZ}\sigma}_{U}^{2}\sigma_{Z}^{2} & \beta_{RU}^{2}\sigma_{U}^{4} \end{matrix} \right)$

Hence, $\beta_{YX|R=r}$ = $\frac{Cov(X,Y|R=r)}{Var(X|R=r)}$ = $\frac{{\beta_{YX}\sigma}_{X}^{2}}{\sigma_{X}^{2}}$ = $\beta_{YX}$, which means that both CRA and MI estimators are unbiased when *Y* is partially observed.

However, note that $\beta_{XY|R=r}$ = $\frac{Cov(X,Y|R=r)}{Var(Y|R=r)}$

$$= \frac{{\beta_{YX}\sigma}_{X}^{2}}{\beta_{YX}^{2}\sigma_{X}^{2}+{\beta_{YU}^{2}\sigma}_{U}^{2}+\sigma_{Y}^{2} -\beta_{YU}^{2}\beta_{RU}^{2}\sigma_{U}^{4}/Var(R)}$$

$$= \frac{{\beta_{YX}\sigma}_{X}^{2}}{\beta_{YX}^{2}\sigma_{X}^{2}+{\beta_{YU}^{2}\sigma}_{U}^{2}+\sigma_{Y}^{2}}\times\frac{1}{1-\beta_{YU}^{2}\beta_{RU}^{2}\sigma_{U}^{4}/\{\left( \beta_{YX}^{2}\sigma_{X}^{2}+{\beta_{YU}^{2}\sigma}_{U}^{2}+\sigma_{Y}^{2} \right)Var\left( R \right)\}}$$

$$=\beta_{XY} \times\frac{1}{1-\left\{ \beta_{YU}^{2}\beta_{RU}^{2}\sigma_{U}^{4}/\left( \beta_{YX}^{2}\sigma_{X}^{2}+{\beta_{YU}^{2}\sigma}_{U}^{2}+\sigma_{Y}^{2} \right)(\beta_{RZ}^{2}\sigma_{Z}^{2}+\beta_{RU}^{2}\sigma_{U}^{2}+\sigma_{R}^{2}) \right\}}$$

as per Equation 3.1 in the main text.

Using a similar approach, the joint conditional distribution of *Y*, *X*, and *U* given *Z* and *R*, *f*(*Y*, *X*, *U* | *Z*=z, *R*=r), is multivariate normal with covariance matrix **∑****, where

**∑**** = $\left( \begin{matrix} \beta_{YX}^{2}\sigma_{X}^{2}+{\beta_{YU}^{2}\sigma}_{U}^{2}+\sigma_{Y}^{2} & {\beta_{YX}\sigma}_{X}^{2} & {\beta_{YU}\sigma}_{U}^{2} \\ {\beta_{YX}\sigma}_{X}^{2} & \sigma_{X}^{2} & 0 \\ {\beta_{YU}\sigma}_{U}^{2} & 0 & \sigma_{U}^{2} \end{matrix} \right)- \frac{1}{\sigma_{Z}^{2}Var\left( R \right)-{\beta_{RZ}^{2}\sigma}_{Z}^{4}}\times$

$$\left( \begin{matrix} 0 & {\beta_{YU}\beta_{RU}\sigma}_{U}^{2} \\ 0 & 0 \\ 0 & {\beta_{RU}\sigma}_{U}^{2} \end{matrix} \right)\left( \begin{matrix} Var(R) & {-\beta}_{RZ}\sigma_{Z}^{2} \\ {{-\beta}_{RZ}\sigma}_{Z}^{2} & \sigma_{Z}^{2} \end{matrix} \right)\left( \begin{matrix} 0 & 0 & 0 \\ {\beta_{YU}\beta_{RU}\sigma}_{U}^{2} & 0 & \beta_{RU}\sigma_{U}^{2} \end{matrix} \right)$$

= $\left( \begin{matrix} \beta_{YX}^{2}\sigma_{X}^{2}+{\beta_{YU}^{2}\sigma}_{U}^{2}+\sigma_{Y}^{2} & {\beta_{YX}\sigma}_{X}^{2} & {\beta_{YU}\sigma}_{U}^{2} \\ {\beta_{YX}\sigma}_{X}^{2} & \sigma_{X}^{2} & 0 \\ {\beta_{YU}\sigma}_{U}^{2} & 0 & \sigma_{U}^{2} \end{matrix} \right)- \frac{1}{\sigma_{Z}^{2}Var\left( R \right)-{\beta_{RZ}^{2}\sigma}_{Z}^{4}}\times\left( \begin{matrix} 0 & {\beta_{YU}\beta_{RU}\sigma}_{U}^{2} \\ 0 & 0 \\ 0 & {\beta_{RU}\sigma}_{U}^{2} \end{matrix} \right)\left( \begin{matrix} {{-\beta}_{RZ}\beta}_{YU}\beta_{RU}\sigma_{Z}^{2}\sigma_{U}^{2} & 0 & {-\beta}_{RZ}\beta_{RU}\sigma_{Z}^{2}\sigma_{U}^{2} \\ \beta_{YU}\beta_{RU}\sigma_{Z}^{2}\sigma_{U}^{2} & 0 & \beta_{RU}\sigma_{Z}^{2}\sigma_{U}^{2} \end{matrix} \right)$

= $\left( \begin{matrix} \beta_{YX}^{2}\sigma_{X}^{2}+{\beta_{YU}^{2}\sigma}_{U}^{2}+\sigma_{Y}^{2} & {\beta_{YX}\sigma}_{X}^{2} & {\beta_{YU}\sigma}_{U}^{2} \\ {\beta_{YX}\sigma}_{X}^{2} & \sigma_{X}^{2} & 0 \\ {\beta_{YU}\sigma}_{U}^{2} & 0 & \sigma_{U}^{2} \end{matrix} \right)- \frac{1}{\sigma_{Z}^{2}Var\left( R \right)-{\beta_{RZ}^{2}\sigma}_{Z}^{4}}\times\left( \begin{matrix} \beta_{YU}^{2}\beta_{RU}^{2}\sigma_{Z}^{2}\sigma_{U}^{4} & 0 & \beta_{YU}\beta_{RU}^{2}\sigma_{Z}^{2}\sigma_{U}^{4} \\ 0 & 0 & 0 \\ \beta_{YU}\beta_{RU}^{2}\sigma_{Z}^{2}\sigma_{U}^{4} & 0 & \beta_{RU}^{2}\sigma_{Z}^{2}\sigma_{U}^{4} \end{matrix} \right)$

Hence, $\beta_{YX|Z=z,R=r}$ = $\frac{Cov(X,Y|Z=z,R=r)}{Var(X|Z=z,R=r)}$ = $\frac{{\beta_{YX}\sigma}_{X}^{2}}{\sigma_{X}^{2}}$ = $\beta_{YX}$. Therefore, the MI estimator will be unbiased when *Y* is partially observed, regardless of whether *Z* is included in the imputation model for *Y*.

However, $\beta_{XY|Z=z,R=r}$ = $\frac{Cov(X,Y|Z=z,R=r)}{Var(Y|Z=z,R=r)}$

$$= \frac{{\beta_{YX}\sigma}_{X}^{2}}{\beta_{YX}^{2}\sigma_{X}^{2}+{\beta_{YU}^{2}\sigma}_{U}^{2}+\sigma_{Y}^{2} -\beta_{YU}^{2}\beta_{RU}^{2}\sigma_{Z}^{2}\sigma_{U}^{4}/(\sigma_{Z}^{2}Var\left( R \right)-{\beta_{RZ}^{2}\sigma}_{Z}^{4})}$$

$$= \frac{{\beta_{YX}\sigma}_{X}^{2}}{\beta_{YX}^{2}\sigma_{X}^{2}+{\beta_{YU}^{2}\sigma}_{U}^{2}+\sigma_{Y}^{2}}\times\frac{1}{1 -\left\{ \beta_{YU}^{2}\beta_{RU}^{2}\sigma_{U}^{4}/\left( \beta_{YX}^{2}\sigma_{X}^{2}+{\beta_{YU}^{2}\sigma}_{U}^{2}+\sigma_{Y}^{2} \right)\left( \beta_{RZ}^{2}\sigma_{Z}^{2}+\beta_{RU}^{2}\sigma_{U}^{2}+\sigma_{R}^{2}-{\beta_{RZ}^{2}\sigma}_{Z}^{2} \right) \right\}}$$

$$= \beta_{XY}\times\frac{1}{1 -\left\{ \beta_{YU}^{2}\beta_{RU}^{2}\sigma_{U}^{4}/\left( \beta_{YX}^{2}\sigma_{X}^{2}+{\beta_{YU}^{2}\sigma}_{U}^{2}+\sigma_{Y}^{2} \right)\left( \beta_{RU}^{2}\sigma_{U}^{2}+\sigma_{R}^{2} \right) \right\}}$$

as per Equation 3.2 in the main text.

Note that $\beta_{XY|R=r}$ and $\beta_{XY|Z=z,R=r}$ do not depend on the specific values of *r* and *z*, hence, as before, we use the more general forms $\beta_{XY|R}$ and $\beta_{XY|Z,R}$ hereafter.

Recall that $\beta_{XY|R}=\beta_{XY} \times\frac{1}{1-\left\{ \beta_{YU}^{2}\beta_{RU}^{2}\sigma_{U}^{4}/\left( \beta_{YX}^{2}\sigma_{X}^{2}+{\beta_{YU}^{2}\sigma}_{U}^{2}+\sigma_{Y}^{2} \right)(\beta_{RZ}^{2}\sigma_{Z}^{2}+\beta_{RU}^{2}\sigma_{U}^{2}+\sigma_{R}^{2}) \right\}}=\beta_{XY} \times\frac{1}{1 - \alpha^{2}}$

where $\alpha^{2}=\frac{\beta_{YU}^{2}\beta_{RU}^{2}\sigma_{U}^{4}}{\left( \beta_{YX}^{2}\sigma_{X}^{2}+{\beta_{YU}^{2}\sigma}_{U}^{2}+\sigma_{Y}^{2} \right)(\beta_{RZ}^{2}\sigma_{Z}^{2}+\beta_{RU}^{2}\sigma_{U}^{2}+\sigma_{R}^{2})}=\frac{\beta_{YU}^{2}\beta_{RU}^{2}\sigma_{U}^{4}}{\beta_{YU}^{2}\beta_{RU}^{2}\sigma_{U}^{4} + other positive terms}$

Since 0 < $\alpha^{2}$ < 1 (assuming Cov(*Y*,*R*) ≠ 0), $\left| \beta_{XY|R} \right|$ is always greater than $\left| \beta_{XY} \right|$.

Furthermore, $\beta_{XY|R,Z}=\beta_{XY}\times\frac{1}{1 - \tau^{2}\alpha^{2}}$ where $\alpha^{2}$ is defined as above and $\tau^{2}=\frac{\beta_{RZ}^{2}\sigma_{Z}^{2}+\beta_{RU}^{2}\sigma_{U}^{2}+\sigma_{R}^{2}}{\beta_{RU}^{2}\sigma_{U}^{2}+\sigma_{R}^{2}}$

Since $\tau^{2}$ > 1 (assuming Cov(*Z*,*R*) ≠ 0), $\tau^{2}\alpha^{2}$ > $\alpha^{2}$ > 0.

Also, $\tau^{2}\alpha^{2}=$ $\frac{\beta_{YU}^{2}\beta_{RU}^{2}\sigma_{U}^{4}}{\left( \beta_{YX}^{2}\sigma_{X}^{2}+{\beta_{YU}^{2}\sigma}_{U}^{2}+\sigma_{Y}^{2} \right)(\beta_{RZ}^{2}\sigma_{Z}^{2}+\beta_{RU}^{2}\sigma_{U}^{2}+\sigma_{R}^{2})} \times$ $\frac{\beta_{RZ}^{2}\sigma_{Z}^{2}+\beta_{RU}^{2}\sigma_{U}^{2}+\sigma_{R}^{2}}{\beta_{RU}^{2}\sigma_{U}^{2}+\sigma_{R}^{2}}$

$=\frac{\beta_{YU}^{2}\beta_{RU}^{2}\sigma_{U}^{4}}{\left( \beta_{YX}^{2}\sigma_{X}^{2}+{\beta_{YU}^{2}\sigma}_{U}^{2}+\sigma_{Y}^{2} \right)(\beta_{RU}^{2}\sigma_{U}^{2}+\sigma_{R}^{2})}=$ $\frac{\beta_{YU}^{2}\beta_{RU}^{2}\sigma_{U}^{4}}{\beta_{YU}^{2}\beta_{RU}^{2}\sigma_{U}^{4} + other positive terms}$ < 1

Therefore, 0 < $\alpha^{2}$ < $\tau^{2}\alpha^{2}$ < 1, and hence,$\left| \beta_{XY|R,Z} \right|$ > $\left| \beta_{XY|R} \right|$ > $\left| \beta_{XY} \right|$ and so bias of the *Y* coefficient will be amplified when *Z* is also included as a predictor in the imputation model for *X* (as well as *Y*) in the main text.

**Verification of theoretical expressions**

Using the same approach as in Section S2, the theoretical expressions for $\beta_{XY|R}$ and $\beta_{XY|R,Z}$ were verified using simulation. We also verified that $\beta_{YX|R}=\beta_{YX|R,Z}=$ $\beta_{YX}$ when *Y* was partially observed (*i.e.* that there was no bias using either imputation model in this setting). We used 1000 simulations, and each simulated dataset contained 100,000 observations. In each simulated dataset, the values of each coefficient ($\beta_{YX}$, $\beta_{RZ}$, *etc*.) and each error variance ($\sigma_{X}^{2}$, $\sigma_{Z}^{2}$, *etc.*) were sampled from a uniform distribution *U*(0, 2). For simplicity, $\mu_{X}$, $\mu_{Z}$, and $\mu_{U}$ were set equal to zero (note that the equations do not depend on these parameters). Data were then generated using the models for *Y*, *X*, *Z*, *U*, and *R*, specified above. $\beta_{XY|R}$ and $\beta_{XY|R,Z}$ were calculated using the theoretical expressions above. They were also estimated empirically by calculating the *Y* coefficient when fitting a linear regression of (i) *X* on *Y*, conditional on *R*, and (ii) *X* on *Y*, conditional on *R* and *Z.* Similarly, the bias of $\beta_{YX|R}$ and $\beta_{YX|R,Z}$ were estimated empirically by calculating the difference in the *X* coefficient when fitting a linear regression of (i) *Y* on *X*, (ii) *Y* on *X*, conditional on *R*, and (iii) *Y* on *X*, conditional on *R* and *Z*.

The median difference between the theoretical and empirical values of $\beta_{XY|R}$ and $\beta_{XY|R,Z}$ was 0.000 (5^th^ – 95^th^ percentile: -0.002 - 0.002) and 0.000 (5^th^ – 95^th^ percentile: -0.002 - 0.003), respectively. The median value of the bias of $\beta_{YX|R}$ and $\beta_{YX|R,Z}$ was 0.000 (5^th^ – 95^th^ percentile: -0.008 - 0.007) and 0.000 (5^th^ – 95^th^ percentile: -0.010 - 0.010), respectively. Therefore, we conclude that the theoretical expressions are correct.

*Section S5. Scenario 3. Derivation of the theoretical expression for the maximum additional bias when a continuous outcome Y or a continuous exposure X is partially observed, with missingness caused by Y and X, when the imputation model includes an auxiliary variable Z that predicts missingness but not the missing values*

As per the main text, in Scenario 3 we assume that the joint distribution of *Y*, *X*, *Z*, and *R*, *f*(*Y*, *X*, *Z*, R), is multivariate normal, with mean **µ**, covariance matrix **∑**, with associated univariate normal distributions defined as follows: *Y* = $\beta_{YX}$*X* + $\varepsilon_{Y}$, where $\varepsilon_{Y}$ ~ N(0, $\sigma_{Y}^{2}$); *X* ~ N($\mu_{X}$, $\sigma_{X}^{2}$); *Z* ~ N($\mu_{Z}$, $\sigma_{Z}^{2}$); and *R* = $\beta_{RY}$*Y* + $\beta_{RX}$*X* + $\beta_{RZ}$*Z* + $\varepsilon_{R}$, where $\varepsilon_{R}$ ~ N(0, $\sigma_{R}^{2}$).

We once again assume that each of *Y* and *R* is a linear combination of the variables causing it plus an error term (with *X* and *Z* having no direct causes), with no interactions, all errors uncorrelated, no model mis-specification, and no measurement error, and that an ordinary least squares (OLS) estimator is used to obtain estimates in both analysis and imputation models.

Hence,

**µ** = $\left( \begin{aligned} \beta_{YX} \mu_{X} \\ \mu_{X} \\ \mu_{Z} \\ \beta_{RY}\beta_{YX} \mu_{X}+\beta_{RX} \mu_{X}+\beta_{RZ} \mu_{Z} \end{aligned} \right)$

and

**∑** = $\left( \begin{matrix} \beta_{YX}^{2}\sigma_{X}^{2}+\sigma_{Y}^{2} & {\beta_{YX}\sigma}_{X}^{2} & 0 & \beta_{RY}(\beta_{YX}^{2}\sigma_{X}^{2}+\sigma_{Y}^{2})+\beta_{YX}\beta_{RX}\sigma_{X}^{2} \\ {\beta_{YX}\sigma}_{X}^{2} & \sigma_{X}^{2} & 0 & {(\beta_{YX}\beta_{RY}+\beta_{RX})\sigma}_{X}^{2} \\ 0 & 0 & \sigma_{Z}^{2} & {\beta_{RZ}\sigma}_{Z}^{2} \\ \beta_{RY}(\beta_{YX}^{2}\sigma_{X}^{2}+\sigma_{Y}^{2})+\beta_{YX}\beta_{RX}\sigma_{X}^{2} & {(\beta_{YX}\beta_{RY}+\beta_{RX})\sigma}_{X}^{2} & {\beta_{RZ}\sigma}_{Z}^{2} & Var(R) \end{matrix} \right)$

where $Var(R)$ = $\beta_{RY}^{2}\left( \beta_{YX}^{2}\sigma_{X}^{2}+\sigma_{Y}^{2} \right)+2\beta_{YX}\beta_{RY}\beta_{RX}\sigma_{X}^{2}+\beta_{RX}^{2}\sigma_{X}^{2}+\beta_{RZ}^{2}\sigma_{Z}^{2}+\sigma_{R}^{2}$

**Derivation of maximum additional bias equations in terms of the direct effect sizes**

We use the same general expressions for the magnitude of the maximum bias of both the MI estimator of $\beta_{YX}$, and the estimator of the *Y* coefficient from the imputation model for *X*, as in previous sections, namely, $\beta_{YX|Z,R}-\beta_{YX|R}$ and $\beta_{XY|Z,R}-\beta_{XY|R}$, respectively.

We can express $\beta_{YX|R=r}$, $\beta_{XY|R=r}$, $\beta_{XY|Z=z,R=r}$, and $\beta_{XY|Z=z,R=r}$ in terms of the direct effect sizes and error variances using standard results.

The joint conditional distribution of *Y*, *X*, and *Z*, given *R*, *f*(*Y*, *X*, *Z* | *R*=r), is multivariate normal with covariance matrix **∑***, where:

**∑*** = $\left( \begin{matrix} \beta_{YX}^{2}\sigma_{X}^{2}+\sigma_{Y}^{2} & {\beta_{YX}\sigma}_{X}^{2} & 0 \\ {\beta_{YX}\sigma}_{X}^{2} & \sigma_{X}^{2} & 0 \\ 0 & 0 & \sigma_{Z}^{2} \end{matrix} \right)- \frac{1}{Var(R)}\times\left( \begin{matrix} \beta_{RY}(\beta_{YX}^{2}\sigma_{X}^{2}+\sigma_{Y}^{2})+\beta_{YX}\beta_{RX}\sigma_{X}^{2} \\ {(\beta_{YX}\beta_{RY}+\beta_{RX})\sigma}_{X}^{2} \\ {\beta_{RZ}\sigma}_{Z}^{2} \end{matrix} \right)\left( \begin{matrix} \beta_{RY}(\beta_{YX}^{2}\sigma_{X}^{2}+\sigma_{Y}^{2})+\beta_{YX}\beta_{RX}\sigma_{X}^{2} & {(\beta_{YX}\beta_{RY}+\beta_{RX})\sigma}_{X}^{2} & \beta_{RZ}\sigma_{Z}^{2} \end{matrix} \right)$

= $\left( \begin{matrix} \beta_{YX}^{2}\sigma_{X}^{2}+\sigma_{Y}^{2} & {\beta_{YX}\sigma}_{X}^{2} & 0 \\ {\beta_{YX}\sigma}_{X}^{2} & \sigma_{X}^{2} & 0 \\ 0 & 0 & \sigma_{Z}^{2} \end{matrix} \right)- \frac{1}{Var(R)}\times\left( \begin{matrix} {\{\beta_{RY}(\beta_{YX}^{2}\sigma_{X}^{2}+\sigma_{Y}^{2})+\beta_{YX}\beta_{RX}\sigma_{X}^{2}\}}^{2} & \ldots& \ldots\\ (\beta_{YX}\beta_{RY}+\beta_{RX})\{\beta_{RY}(\beta_{YX}^{2}\sigma_{X}^{2}+\sigma_{Y}^{2})+\beta_{YX}\beta_{RX}\sigma_{X}^{2}\}\sigma_{X}^{2} & {(\beta_{YX}\beta_{RY}+\beta_{RX})}^{2}\sigma_{X}^{4} & \ldots\\ \{\beta_{RY}(\beta_{YX}^{2}\sigma_{X}^{2}+\sigma_{Y}^{2})+\beta_{YX}\beta_{RX}\sigma_{X}^{2}\}\beta_{RZ}\sigma_{Z}^{2} & (\beta_{YX}\beta_{RY}+\beta_{RX}){\beta_{RZ}\sigma}_{X}^{2}\sigma_{Z}^{2} & \beta_{RZ}^{2}\sigma_{Z}^{4} \end{matrix} \right)$

and the joint conditional distribution of *Y* and *X*, given *Z* and *R*, *f*(*Y*, *X* | *Z*=z, *R*=r), is multivariate normal with covariance matrix **∑****, where:

**∑**** = $\left( \begin{matrix} \beta_{YX}^{2}\sigma_{X}^{2}+\sigma_{Y}^{2} & {\beta_{YX}\sigma}_{X}^{2} \\ {\beta_{YX}\sigma}_{X}^{2} & \sigma_{X}^{2} \end{matrix} \right)- \frac{1}{\sigma_{Z}^{2}Var\left( R \right)-{\beta_{RZ}^{2}\sigma}_{Z}^{4}}\times\left( \begin{matrix} 0 & \beta_{RY}(\beta_{YX}^{2}\sigma_{X}^{2}+\sigma_{Y}^{2})+\beta_{YX}\beta_{RX}\sigma_{X}^{2} \\ 0 & {(\beta_{YX}\beta_{RY}+\beta_{RX})\sigma}_{X}^{2} \end{matrix} \right)\left( \begin{matrix} Var(R) & {-\beta}_{RZ}\sigma_{Z}^{2} \\ {{-\beta}_{RZ}\sigma}_{Z}^{2} & \sigma_{Z}^{2} \end{matrix} \right)\left( \begin{matrix} 0 & 0 \\ \beta_{RY}(\beta_{YX}^{2}\sigma_{X}^{2}+\sigma_{Y}^{2})+\beta_{YX}\beta_{RX}\sigma_{X}^{2} & {(\beta_{YX}\beta_{RY}+\beta_{RX})\sigma}_{X}^{2} \end{matrix} \right)$

= $\left( \begin{matrix} \beta_{YX}^{2}\sigma_{X}^{2}+\sigma_{Y}^{2} & {\beta_{YX}\sigma}_{X}^{2} \\ {\beta_{YX}\sigma}_{X}^{2} & \sigma_{X}^{2} \end{matrix} \right)- \frac{1}{\sigma_{Z}^{2}Var\left( R \right)-{\beta_{RZ}^{2}\sigma}_{Z}^{4}}\times$

$$\left( \begin{matrix} 0 & \beta_{RY}(\beta_{YX}^{2}\sigma_{X}^{2}+\sigma_{Y}^{2})+\beta_{YX}\beta_{RX}\sigma_{X}^{2} \\ 0 & {(\beta_{YX}\beta_{RY}+\beta_{RX})\sigma}_{X}^{2} \end{matrix} \right)\left( \begin{matrix} -\{\beta_{RY}(\beta_{YX}^{2}\sigma_{X}^{2}+\sigma_{Y}^{2})+\beta_{YX}\beta_{RX}\sigma_{X}^{2}\}\beta_{RZ}\sigma_{Z}^{2} & -\beta_{RZ}(\beta_{YX}\beta_{RY}+\beta_{RX})\sigma_{X}^{2}\sigma_{Z}^{2} \\ \{\beta_{RY}(\beta_{YX}^{2}\sigma_{X}^{2}+\sigma_{Y}^{2})+\beta_{YX}\beta_{RX}\sigma_{X}^{2}\}\sigma_{Z}^{2} & {(\beta_{YX}\beta_{RY}+\beta_{RX})\sigma}_{X}^{2}\sigma_{Z}^{2} \end{matrix} \right)$$

= $\left( \begin{matrix} \beta_{YX}^{2}\sigma_{X}^{2}+\sigma_{Y}^{2} & {\beta_{YX}\sigma}_{X}^{2} \\ {\beta_{YX}\sigma}_{X}^{2} & \sigma_{X}^{2} \end{matrix} \right)- \frac{1}{\sigma_{Z}^{2}Var\left( R \right)-{\beta_{RZ}^{2}\sigma}_{Z}^{4}}\times\left( \begin{matrix} \left( \beta_{RY}(\beta_{YX}^{2}\sigma_{X}^{2}+\sigma_{Y}^{2})+\beta_{YX}\beta_{RX}\sigma_{X}^{2} \right)^{2}\sigma_{Z}^{2} & (\beta_{RY}(\beta_{YX}^{2}\sigma_{X}^{2}+\sigma_{Y}^{2})+\beta_{YX}\beta_{RX}\sigma_{X}^{2}){(\beta_{YX}\beta_{RY}+\beta_{RX})\sigma}_{X}^{2}\sigma_{Z}^{2} \\ (\beta_{RY}(\beta_{YX}^{2}\sigma_{X}^{2}+\sigma_{Y}^{2})+\beta_{YX}\beta_{RX}\sigma_{X}^{2}){(\beta_{YX}\beta_{RY}+\beta_{RX})\sigma}_{X}^{2}\sigma_{Z}^{2} & {{(\beta_{YX}\beta_{RY}+\beta_{RX})}^{2}\sigma}_{X}^{4}\sigma_{Z}^{2} \end{matrix} \right)$

As per the main text, we consider the bias of the MI estimator in two separate settings where: (i) *Y* is partially observed, and (ii) *X* is partially observed.

**Setting 1: *Y* is partially observed**

Using the results above, $\beta_{YX|R=r}$ = $\frac{Cov(X,Y|R=r)}{Var(X|R=r)}$

$$=\frac{{\beta_{YX}\sigma}_{X}^{2}Var(R)- (\beta_{YX}\beta_{RY}+\beta_{RX})\{\beta_{RY}(\beta_{YX}^{2}\sigma_{X}^{2}+\sigma_{Y}^{2})+\beta_{YX}\beta_{RX}\sigma_{X}^{2}\}\sigma_{X}^{2}}{\sigma_{X}^{2}Var(R)-{(\beta_{YX}\beta_{RY}+\beta_{RX})}^{2}\sigma_{X}^{4}}$$

$$=\frac{\beta_{YX}\{\beta_{RY}^{2}\left( \beta_{YX}^{2}\sigma_{X}^{2}+\sigma_{Y}^{2} \right)+2\beta_{RY}\beta_{RX}{\beta_{YX}\sigma}_{X}^{2}+\beta_{RX}^{2}\sigma_{X}^{2}+\beta_{RZ}^{2}\sigma_{Z}^{2}+\sigma_{R}^{2}\}}{\beta_{RY}^{2}\left( \beta_{YX}^{2}\sigma_{X}^{2}+\sigma_{Y}^{2} \right)+2\beta_{YX}\beta_{RY}\beta_{RX}\sigma_{X}^{2}+\beta_{RX}^{2}\sigma_{X}^{2}+\beta_{RZ}^{2}\sigma_{Z}^{2}+\sigma_{R}^{2}-{(\beta_{YX}\beta_{RY}+\beta_{RX})}^{2}\sigma_{X}^{2}}-\left\{ \frac{(\beta_{YX}\beta_{RY}+\beta_{RX})\{\beta_{RY}(\beta_{YX}^{2}\sigma_{X}^{2}+\sigma_{Y}^{2})+\beta_{YX}\beta_{RX}\sigma_{X}^{2}\}}{\beta_{RY}^{2}\left( \beta_{YX}^{2}\sigma_{X}^{2}+\sigma_{Y}^{2} \right)+2\beta_{YX}\beta_{RY}\beta_{RX}\sigma_{X}^{2}+\beta_{RX}^{2}\sigma_{X}^{2}+\beta_{RZ}^{2}\sigma_{Z}^{2}+\sigma_{R}^{2}-{(\beta_{YX}\beta_{RY}+\beta_{RX})}^{2}\sigma_{X}^{2}} \right\}$$

$$=\frac{\beta_{YX}\beta_{RY}^{2}\left( \beta_{YX}^{2}\sigma_{X}^{2}+\sigma_{Y}^{2} \right)+2\beta_{YX}\beta_{RY}\beta_{RX}{\beta_{YX}\sigma}_{X}^{2}+\beta_{YX}\beta_{RX}^{2}\sigma_{X}^{2}+\beta_{YX}\beta_{RZ}^{2}\sigma_{Z}^{2}+\beta_{YX}\sigma_{R}^{2}}{\beta_{YX}^{2}\beta_{RY}^{2}\sigma_{X}^{2}+\beta_{RY}^{2}\sigma_{Y}^{2}+2\beta_{YX}\beta_{RY}\beta_{RX}\sigma_{X}^{2}+\beta_{RX}^{2}\sigma_{X}^{2}+\beta_{RZ}^{2}\sigma_{Z}^{2}+\sigma_{R}^{2}-\beta_{YX}^{2}\beta_{RY}^{2}\sigma_{X}^{2}-\beta_{RX}^{2}\sigma_{X}^{2}-2\beta_{YX}\beta_{RY}\beta_{RX}\sigma_{X}^{2}}-\left\{ \frac{\beta_{YX}\beta_{RY}^{2}\left( \beta_{YX}^{2}\sigma_{X}^{2}+\sigma_{Y}^{2} \right)+\beta_{RX}\beta_{RY}(\beta_{YX}^{2}\sigma_{X}^{2}+\sigma_{Y}^{2})+\beta_{YX}^{2}\beta_{RY}\beta_{RX}\sigma_{X}^{2}+\beta_{YX}\beta_{RX}^{2}\sigma_{X}^{2}\}}{\beta_{YX}^{2}\beta_{RY}^{2}\sigma_{X}^{2}+\beta_{RY}^{2}\sigma_{Y}^{2}+2\beta_{YX}\beta_{RY}\beta_{RX}\sigma_{X}^{2}+\beta_{RX}^{2}\sigma_{X}^{2}+\beta_{RZ}^{2}\sigma_{Z}^{2}+\sigma_{R}^{2}-\beta_{YX}^{2}\beta_{RY}^{2}\sigma_{X}^{2}-\beta_{RX}^{2}\sigma_{X}^{2}-2\beta_{YX}\beta_{RY}\beta_{RX}\sigma_{X}^{2}} \right\}$$

$$= \frac{\beta_{YX}\beta_{RZ}^{2}\sigma_{Z}^{2}+\beta_{YX}\sigma_{R}^{2}-\beta_{RY}\beta_{RX}\sigma_{Y}^{2}}{\beta_{RY}^{2}\sigma_{Y}^{2}+\beta_{RZ}^{2}\sigma_{Z}^{2}+\sigma_{R}^{2}}$$

$$= \beta_{YX} \times\left\{ \frac{\beta_{RZ}^{2}\sigma_{Z}^{2}+\sigma_{R}^{2}-\frac{\beta_{RY}\beta_{RX}\sigma_{Y}^{2}}{\beta_{YX}}}{\beta_{RY}^{2}\sigma_{Y}^{2}+\sigma_{R}^{2}+\beta_{RZ}^{2}\sigma_{Z}^{2}} \right\}$$

$$= \beta_{YX} \times\left\{ 1-\frac{\left( \beta_{RY}+\frac{\beta_{RX}}{\beta_{YX}} \right)\beta_{RY}\sigma_{Y}^{2}}{\beta_{RY}^{2}\sigma_{Y}^{2}+\sigma_{R}^{2}+\beta_{RZ}^{2}\sigma_{Z}^{2}} \right\}$$

Similarly, the *X* coefficient from the imputation model for *Y* when *X* and *Z* are used as predictors tends to $\beta_{YX|Z,R}$ as the proportion of missing data tends to one, where:

$\beta_{YX|Z=z,R=r}$ = $\frac{Cov(X,Y|Z=z,R=r)}{Var(X|Z=z,R=r)}$

$$=\frac{{\beta_{YX}\sigma}_{X}^{2}\left( \sigma_{Z}^{2}Var\left( R \right)-{\beta_{RZ}^{2}\sigma}_{Z}^{4} \right)-(\beta_{RY}(\beta_{YX}^{2}\sigma_{X}^{2}+\sigma_{Y}^{2})+\beta_{YX}\beta_{RX}\sigma_{X}^{2}){(\beta_{YX}\beta_{RY}+\beta_{RX})\sigma}_{X}^{2}\sigma_{Z}^{2}}{\sigma_{X}^{2}\left( \sigma_{Z}^{2}Var\left( R \right)-{\beta_{RZ}^{2}\sigma}_{Z}^{4} \right)-{{(\beta_{YX}\beta_{RY}+\beta_{RX})}^{2}\sigma}_{X}^{4}\sigma_{Z}^{2}}$$

$$=\frac{{\beta_{YX}\sigma}_{X}^{2}\sigma_{Z}^{2}\left\{ \beta_{RY}^{2}\left( \beta_{YX}^{2}\sigma_{X}^{2}+\sigma_{Y}^{2} \right)+2\beta_{RY}\beta_{RX}{\beta_{YX}\sigma}_{X}^{2}+\beta_{RX}^{2}\sigma_{X}^{2}+\beta_{RZ}^{2}\sigma_{Z}^{2}+\sigma_{R}^{2}-{\beta_{RZ}^{2}\sigma}_{Z}^{2} \right\}}{\sigma_{X}^{2}\sigma_{Z}^{2}\left( \beta_{RY}^{2}\left( \beta_{YX}^{2}\sigma_{X}^{2}+\sigma_{Y}^{2} \right)+2\beta_{RY}\beta_{RX}{\beta_{YX}\sigma}_{X}^{2}+\beta_{RX}^{2}\sigma_{X}^{2}+\beta_{RZ}^{2}\sigma_{Z}^{2}+\sigma_{R}^{2}-{\beta_{RZ}^{2}\sigma}_{Z}^{2}-{{(\beta_{YX}\beta_{RY}+\beta_{RX})}^{2}\sigma}_{X}^{2} \right)}-$$

$$\left\{ \frac{\sigma_{X}^{2}\sigma_{Z}^{2}(\beta_{RY}(\beta_{YX}^{2}\sigma_{X}^{2}+\sigma_{Y}^{2})+\beta_{YX}\beta_{RX}\sigma_{X}^{2})(\beta_{YX}\beta_{RY}+\beta_{RX})}{\sigma_{X}^{2}\sigma_{Z}^{2}\left( \beta_{RY}^{2}\left( \beta_{YX}^{2}\sigma_{X}^{2}+\sigma_{Y}^{2} \right)+2\beta_{RY}\beta_{RX}{\beta_{YX}\sigma}_{X}^{2}+\beta_{RX}^{2}\sigma_{X}^{2}+\beta_{RZ}^{2}\sigma_{Z}^{2}+\sigma_{R}^{2}-{\beta_{RZ}^{2}\sigma}_{Z}^{2}-{{(\beta_{YX}\beta_{RY}+\beta_{RX})}^{2}\sigma}_{X}^{2} \right)} \right\}$$

$$=\frac{\beta_{YX}\beta_{RY}^{2}\left( \beta_{YX}^{2}\sigma_{X}^{2}+\sigma_{Y}^{2} \right)+2\beta_{YX}^{2}\beta_{RY}\beta_{RX}\sigma_{X}^{2}+\beta_{YX}\beta_{RX}^{2}\sigma_{X}^{2}+\beta_{YX}\sigma_{R}^{2}}{\beta_{RY}^{2}\sigma_{Y}^{2}+\sigma_{R}^{2}}-$$

$$\left\{ \frac{\beta_{YX}\beta_{RY}^{2}\left( \beta_{YX}^{2}\sigma_{X}^{2}+\sigma_{Y}^{2} \right)+2\beta_{YX}^{2}\beta_{RY}\beta_{RX}\sigma_{X}^{2}+\beta_{YX}\beta_{RX}^{2}\sigma_{X}^{2}+\beta_{RY}\beta_{RX}\sigma_{Y}^{2}}{\beta_{RY}^{2}\sigma_{Y}^{2}+\sigma_{R}^{2}} \right\}$$

$$=\beta_{YX}\times\left\{ \frac{\sigma_{R}^{2}-\frac{\beta_{RY}\beta_{RX}\sigma_{Y}^{2}}{\beta_{YX}}}{\beta_{RY}^{2}\sigma_{Y}^{2}+\sigma_{R}^{2}} \right\}$$

$$=\beta_{YX}\times\left\{ 1-\frac{\beta_{RY}\sigma_{Y}^{2}\left( \beta_{RY}+\frac{\beta_{RX}}{\beta_{YX}} \right)}{\beta_{RY}^{2}\sigma_{Y}^{2}+\sigma_{R}^{2}} \right\}$$

Thus, the maximum bias of the MI estimator due to *Y* being MNAR (using only *X* as a predictor in the imputation model for *Y*) is $-\frac{\beta_{YX}\beta_{RY}\sigma_{Y}^{2}\left( \beta_{RY}+\frac{\beta_{RX}}{\beta_{YX}} \right)}{\beta_{RY}^{2}\sigma_{Y}^{2}+\sigma_{R}^{2}+\beta_{RZ}^{2}\sigma_{Z}^{2}}$. The maximum additional bias of the MI estimator (*i.e.* in addition to the bias due to *Y* being MNAR) from including *Z* as a predictor in the imputation model is $\beta_{YX}\beta_{RY}\sigma_{Y}^{2}\left( \beta_{RY}+\frac{\beta_{RX}}{\beta_{YX}} \right)\times\left\{ \frac{1}{\beta_{RY}^{2}\sigma_{Y}^{2}+\sigma_{R}^{2}+\beta_{RZ}^{2}\sigma_{Z}^{2}}-\frac{1}{\beta_{RY}^{2}\sigma_{Y}^{2}+\sigma_{R}^{2}} \right\}=\frac{-\beta_{YX}\beta_{RY}\beta_{RZ}^{2}\sigma_{Y}^{2}\sigma_{Z}^{2}\left( \beta_{RY}+\frac{\beta_{RX}}{\beta_{YX}} \right)}{\left( \beta_{RY}^{2}\sigma_{Y}^{2}+\sigma_{R}^{2}+\beta_{RZ}^{2}\sigma_{Z}^{2} \right)\left( \beta_{RY}^{2}\sigma_{Y}^{2}+\sigma_{R}^{2} \right)}$ as per Equation 4.2 in the main text.

Or in other words, if bias amplification is defined as the bias of $\beta_{YX|Z,R}$ divided by the bias of $\beta_{YX|R}$ (again noting that these expressions do not depend on the specific values of *r* and *z*, and hence we can use the more general forms hereafter), then the maximum bias amplification is: $\frac{-\beta_{YX}\beta_{RY}\sigma_{Y}^{2}\left( \beta_{RY}+\frac{\beta_{RX}}{\beta_{YX}} \right)}{\beta_{RY}^{2}\sigma_{Y}^{2}+\sigma_{R}^{2}}/\frac{-\beta_{YX}\beta_{RY}\sigma_{Y}^{2}\left( \beta_{RY}+\frac{\beta_{RX}}{\beta_{YX}} \right)}{\beta_{RZ}^{2}\sigma_{Z}^{2}+\beta_{RY}^{2}\sigma_{Y}^{2}+\sigma_{R}^{2}}=\frac{\beta_{RZ}^{2}\sigma_{Z}^{2}+\beta_{RY}^{2}\sigma_{Y}^{2}+\sigma_{R}^{2}}{\beta_{RY}^{2}\sigma_{Y}^{2}+\sigma_{R}^{2}}=1+\frac{\beta_{RZ}^{2}\sigma_{Z}^{2}}{\beta_{RY}^{2}\sigma_{Y}^{2}+\sigma_{R}^{2}}$, that is, the maximum bias due to *Y* being MNAR is amplified by a factor of $\left\{ 1+\frac{\beta_{RZ}^{2}\sigma_{Z}^{2}}{\beta_{RY}^{2}\sigma_{Y}^{2}+\sigma_{R}^{2}} \right\}$ when *Z* is included in the imputation model for *Y*.

**Setting 2: *X* is partially observed**

Using the results above,

$\beta_{XY|R=r}$ = $\frac{Cov(X,Y|R=r)}{Var(Y|R=r)}$

$$=\frac{{\beta_{YX}\sigma}_{X}^{2}Var(R)- (\beta_{YX}\beta_{RY}+\beta_{RX})\{\beta_{RY}(\beta_{YX}^{2}\sigma_{X}^{2}+\sigma_{Y}^{2})+\beta_{YX}\beta_{RX}\sigma_{X}^{2}\}\sigma_{X}^{2}}{\left( \beta_{YX}^{2}\sigma_{X}^{2}+\sigma_{Y}^{2} \right)Var(R)-{\{\beta_{RY}(\beta_{YX}^{2}\sigma_{X}^{2}+\sigma_{Y}^{2})+\beta_{YX}\beta_{RX}\sigma_{X}^{2}\}}^{2}}$$

Noting ${\{\beta_{RY}(\beta_{YX}^{2}\sigma_{X}^{2}+\sigma_{Y}^{2})+\beta_{YX}\beta_{RX}\sigma_{X}^{2}\}}^{2}=\beta_{RY}^{2}\left( \beta_{YX}^{2}\sigma_{X}^{2}+\sigma_{Y}^{2} \right)^{2}+\beta_{YX}^{2}\beta_{RX}^{2}\sigma_{X}^{4}+2\beta_{YX}\beta_{RY}\beta_{RX}\sigma_{X}^{2}(\beta_{YX}^{2}\sigma_{X}^{2}+\sigma_{Y}^{2})$, this expression can be simplified to:

$$\beta_{XY|R=r}=\frac{\beta_{YX}\sigma_{X}^{2}\{\beta_{RZ}^{2}\sigma_{Z}^{2}+\sigma_{R}^{2}-\frac{\beta_{RY}\beta_{RX}\sigma_{Y}^{2}}{\beta_{YX}}\}}{\left( \beta_{YX}^{2}\sigma_{X}^{2}+\sigma_{Y}^{2} \right)\left( \beta_{RX}^{2}\sigma_{X}^{2}+\beta_{RZ}^{2}\sigma_{Z}^{2}+\sigma_{R}^{2} \right)-\beta_{YX}^{2}\beta_{RX}^{2}\sigma_{X}^{4}}$$

$$=\frac{\beta_{YX}\sigma_{X}^{2}\{\beta_{RZ}^{2}\sigma_{Z}^{2}+\sigma_{R}^{2}-\frac{\beta_{RY}\beta_{RX}\sigma_{Y}^{2}}{\beta_{YX}}\}}{\left( \beta_{YX}^{2}\sigma_{X}^{2}+\sigma_{Y}^{2} \right)\left( \beta_{RX}^{2}\sigma_{X}^{2}+\beta_{RZ}^{2}\sigma_{Z}^{2}+\sigma_{R}^{2} \right)-\beta_{YX}^{2}\beta_{RX}^{2}\sigma_{X}^{4}}$$

$$=\frac{\beta_{YX}\sigma_{X}^{2}}{\left( \beta_{YX}^{2}\sigma_{X}^{2}+\sigma_{Y}^{2} \right)}\times\frac{\beta_{RZ}^{2}\sigma_{Z}^{2}+\sigma_{R}^{2}-\frac{\beta_{RY}\beta_{RX}\sigma_{Y}^{2}}{\beta_{YX}}}{\beta_{RX}^{2}\sigma_{X}^{2}+\beta_{RZ}^{2}\sigma_{Z}^{2}+\sigma_{R}^{2}-\left\{ \beta_{YX}^{2}\beta_{RX}^{2}\sigma_{X}^{4}/\left( \beta_{YX}^{2}\sigma_{X}^{2}+\sigma_{Y}^{2} \right) \right\}}$$

$$=\beta_{XY}\times\left\{ 1-\frac{\beta_{RX}\left\{ \frac{\beta_{RY}\sigma_{Y}^{2}}{\beta_{YX}}+\beta_{RX}\sigma_{X}^{2}\left( 1-\frac{\beta_{YX}^{2}\sigma_{X}^{2}}{\beta_{YX}^{2}\sigma_{X}^{2}+\sigma_{Y}^{2}} \right) \right\}}{\beta_{RX}^{2}\sigma_{X}^{2}+\beta_{RZ}^{2}\sigma_{Z}^{2}+\sigma_{R}^{2}-\left\{ \beta_{YX}^{2}\beta_{RX}^{2}\sigma_{X}^{4}/\left( \beta_{YX}^{2}\sigma_{X}^{2}+\sigma_{Y}^{2} \right) \right\}} \right\}$$

Similarly,

$\beta_{XY|Z=z,R=r}$ = $\frac{Cov(X,Y|Z=z,R=R)}{Var(Y|Z=z,R=r)}$

$$=\frac{{\beta_{YX}\sigma}_{X}^{2}\left( \sigma_{Z}^{2}Var\left( R \right)-{\beta_{RZ}^{2}\sigma}_{Z}^{4} \right)-(\beta_{RY}(\beta_{YX}^{2}\sigma_{X}^{2}+\sigma_{Y}^{2})+\beta_{YX}\beta_{RX}\sigma_{X}^{2}){(\beta_{YX}\beta_{RY}+\beta_{RX})\sigma}_{X}^{2}\sigma_{Z}^{2}}{\left( \beta_{YX}^{2}\sigma_{X}^{2}+\sigma_{Y}^{2} \right)\left( \sigma_{Z}^{2}Var\left( R \right)-{\beta_{RZ}^{2}\sigma}_{Z}^{4} \right)-\left\{ \beta_{RY}(\beta_{YX}^{2}\sigma_{X}^{2}+\sigma_{Y}^{2})+\beta_{YX}\beta_{RX}\sigma_{X}^{2} \right\}^{2}\sigma_{Z}^{2}}$$

Using results above, this expression can be simplified to:

$$\beta_{XY|Z=z,R=r}=\frac{\beta_{YX}\sigma_{X}^{2}\{\sigma_{R}^{2}-\frac{\beta_{RY}\beta_{RX}\sigma_{Y}^{2}}{\beta_{YX}}\}}{\left( \beta_{YX}^{2}\sigma_{X}^{2}+\sigma_{Y}^{2} \right)\left( \beta_{RX}^{2}\sigma_{X}^{2}+\sigma_{R}^{2} \right)-\beta_{YX}^{2}\beta_{RX}^{2}\sigma_{X}^{4}}$$

$$=\frac{\beta_{YX}\sigma_{X}^{2}}{\left( \beta_{YX}^{2}\sigma_{X}^{2}+\sigma_{Y}^{2} \right)}\times\frac{\sigma_{R}^{2}-\frac{\beta_{RY}\beta_{RX}\sigma_{Y}^{2}}{\beta_{YX}}}{\beta_{RX}^{2}\sigma_{X}^{2}+\sigma_{R}^{2}-\left\{ \beta_{YX}^{2}\beta_{RX}^{2}\sigma_{X}^{4}/\left( \beta_{YX}^{2}\sigma_{X}^{2}+\sigma_{Y}^{2} \right) \right\}}$$

$$=\beta_{XY}\times\left\{ 1-\frac{\frac{\beta_{RY}\beta_{RX}\sigma_{Y}^{2}}{\beta_{YX}}+\beta_{RX}^{2}\sigma_{X}^{2}-\left\{ \frac{\beta_{YX}^{2}\beta_{RX}^{2}\sigma_{X}^{4}}{\left( \beta_{YX}^{2}\sigma_{X}^{2}+\sigma_{Y}^{2} \right)} \right\}}{\beta_{RX}^{2}\sigma_{X}^{2}+\sigma_{R}^{2}-\left\{ \beta_{YX}^{2}\beta_{RX}^{2}\sigma_{X}^{4}/\left( \beta_{YX}^{2}\sigma_{X}^{2}+\sigma_{Y}^{2} \right) \right\}} \right\}$$

$$=\beta_{XY}\times\left\{ 1-\frac{\beta_{RX}\left\{ \frac{\beta_{RY}\sigma_{Y}^{2}}{\beta_{YX}}+\beta_{RX}\sigma_{X}^{2}\left( 1-\frac{\beta_{YX}^{2}\sigma_{X}^{2}}{\beta_{YX}^{2}\sigma_{X}^{2}+\sigma_{Y}^{2}} \right) \right\}}{\beta_{RX}^{2}\sigma_{X}^{2}+\sigma_{R}^{2}-\left\{ \beta_{YX}^{2}\beta_{RX}^{2}\sigma_{X}^{4}/\left( \beta_{YX}^{2}\sigma_{X}^{2}+\sigma_{Y}^{2} \right) \right\}} \right\}$$

Thus, the maximum additional bias of the *Y* coefficient in the imputation model for *X* (*i.e.* in addition to the bias due to *X* being MNAR) from including *Z* as a predictor in the imputation model is: $\beta_{XY}\beta_{RX}\left\{ \frac{\beta_{RY}\sigma_{Y}^{2}}{\beta_{YX}}+\beta_{RX}\sigma_{X}^{2}\left( 1-\frac{\beta_{YX}^{2}\sigma_{X}^{2}}{\beta_{YX}^{2}\sigma_{X}^{2}+\sigma_{Y}^{2}} \right) \right\}\times\left\{ \frac{1}{\beta_{RX}^{2}\sigma_{X}^{2}+\beta_{RZ}^{2}\sigma_{Z}^{2}+\sigma_{R}^{2}-\left\{ \beta_{YX}^{2}\beta_{RX}^{2}\sigma_{X}^{4}/\left( \beta_{YX}^{2}\sigma_{X}^{2}+\sigma_{Y}^{2} \right) \right\}}-\frac{1}{\beta_{RX}^{2}\sigma_{X}^{2}+\sigma_{R}^{2}-\left\{ \beta_{YX}^{2}\beta_{RX}^{2}\sigma_{X}^{4}/\left( \beta_{YX}^{2}\sigma_{X}^{2}+\sigma_{Y}^{2} \right) \right\}} \right\}$ as per Equation 4.4 in the main text (again noting that these expressions do not depend on the specific values of *r* and *z*).

**Verification of theoretical expressions**

As before, the theoretical expressions for the maximum bias of $\beta_{YX|R}$ and $\beta_{XY|R}$, as well as the maximum additional bias of $\beta_{YX|Z,R}$ and $\beta_{XY|Z,R}$, were verified using simulation. We used 1000 simulations, and each simulated dataset contained 100,000 observations. In each simulated dataset, the values of each coefficient ($\beta_{YX}$, $\beta_{RZ}$, *etc*.) and each error variance ($\sigma_{X}^{2}$, $\sigma_{Z}^{2}$, *etc.*) were sampled from a uniform distribution *U*(0, 2). For simplicity, $\mu_{X}$ and $\mu_{Z}$ were set equal to zero (note that the equations do not depend on these parameters). Data were then generated using the models for *Y*, *X*, *Z*, and *R* that were specified above. All bias quantities were calculated using the theoretical expressions. They were also estimated empirically by calculating (for Setting 1) the difference in the *X* coefficient when fitting a linear regression of (i) *Y* on *X*, (ii) *Y* on *X*, conditional on *R*, and (iii) *Y* on *X*, conditional on *R* and *Z*, and (for Setting 2) the difference in the *Y* coefficient when fitting a linear regression of (i) *X* on *Y*, (ii) *X* on *Y*, conditional on *R*, and (iii) *X* on *Y*, conditional on *R* and *Z* (with, in each setting, the difference between the coefficient from models (i) and (ii) used to estimate the maximum bias, and the difference between the coefficient from models (ii) and (iii) used to estimate the maximum additional bias).

In Setting 1, the median difference between the theoretical and empirical values of maximum bias and maximum additional bias was 0.000 (5^th^ – 95^th^ percentile: -0.008 - 0.009) and 0.000 (5^th^ – 95^th^ percentile: -0.006 - 0.006), respectively. In Setting 2, the median difference between the theoretical and empirical values of maximum bias and maximum additional bias was 0.000 (5^th^ – 95^th^ percentile: -0.004 - 0.003) and 0.000 (5^th^ – 95^th^ percentile: -0.003 - 0.003), respectively. Therefore, we conclude that all theoretical expressions are correct.

*Section S6. Stata code for equation verification and illustration, and to generate data as per the simulation studies*

*** Scenario 1 ***

* 1. Verification of bias

*Define postfile to store results

tempname simloop

postfile `simloop' int(i) float(b_yx beta_yx beta_yx_cond_r beta_yx_cond_rz theor_bias emp_bias theor_addbias emp_addbias) using "sim_yxzr_scen1.dta", replace

forvalues i=1/1000 {

clear

*SD

local s_z=runiform(0,2)

local s_r=runiform(0,2)

local s_x=runiform(0,2)

local s_y=runiform(0,2)

*beta

local b_yx=runiform(0,2)

local b_ry=runiform(0,2)

local b_rz=runiform(0,2)

*RVs

quietly set obs 100000

gen z=rnormal(0,`s_z')

gen x=rnormal(0,`s_x')

gen y=rnormal(`b_yx'*x,`s_y')

gen r=rnormal(`b_ry'*y + `b_rz'*z,`s_r')

*Estimate beta_YX

quietly regress y x

local beta_yx=e(b)[1,1]

*Estimate beta_YX|R

quietly regress y x r

*Store estimate

local beta_yx_cond_r=e(b)[1,1]

*Estimate beta_YX|R,Z

quietly regress y x r z

*Store estimate

local beta_yx_cond_rz=e(b)[1,1]

*Calculate theoretical and empirical bias and bias amp

local emp_bias = `beta_yx_cond_r' - `beta_yx'

local emp_addbias = `beta_yx_cond_rz' - `beta_yx_cond_r'

local theor_bias=-`b_yx'*`b_ry'^2*`s_y'^2/(`b_ry'^2*`s_y'^2 + `s_r'^2 + `b_rz'^2*`s_z'^2)

local theor_addbias=`b_yx'*`b_ry'^2*`s_y'^2*((1/(`b_ry'^2*`s_y'^2 + `s_r'^2 + `b_rz'^2*`s_z'^2)) - (1/(`b_ry'^2*`s_y'^2 + `s_r'^2)))

post `simloop' (`i') (`b_yx') (`beta_yx') (`beta_yx_cond_r') (`beta_yx_cond_rz') (`theor_bias') (`emp_bias') (`theor_addbias') (`emp_addbias')

}

postclose `simloop'

use sim_yxzr_scen1, clear

gen diff_bias=theor_bias - emp_bias

gen diff_addbias=theor_addbias-emp_addbias

sum diff_bias diff_addbias, detail

*2. Illustration of max additional bias equation

****** Varying the size of coefficients - still use mu_z=0 and mu_u=0 and all error vars = 1*******

*Define postfile to store results

postfile `simloop' float(b_yx b_ry b_rz max_addbias max_totalbias max_biasmnar) using "maxaddbias_illustration_scen1.dta", replace

foreach b_yx of numlist 0 0.25 0.5 0.75 1 {

foreach b_ry of numlist 0 0.25 0.5 0.75 1 {

foreach b_rz of numlist 0 0.25 0.5 0.75 1 {

clear

local max_addbias=`b_yx'*`b_ry'^2*(1/(`b_ry'^2 + `b_rz'^2 + 1) - 1/(`b_ry'^2 + 1))

local max_totalbias=-`b_yx'*`b_ry'^2/(`b_ry'^2 + 1)

local max_biasmnar=-`b_yx'*`b_ry'^2/(`b_ry'^2 + `b_rz'^2 + 1)

post `simloop' (`b_yx') (`b_ry') (`b_rz') (`max_addbias') (`max_totalbias') (`max_biasmnar')

}

}

}

postclose `simloop'

* 3. Illustration of max additional bias - Binary Y

****** Varying the size of coefficients - still use mu_z=0 and mu_u=0 and all error vars = 1*******

tempname simloop

postfile `simloop' int(nsim) float(b_yx b_ry b_rz beta_x_x se_x_x beta_x_xz se_x_xz) using "MIaddbias_illustration_scen1Ybin.dta", replace

*Create a temporary file for storing simulated data

tempfile tmpfull

forvalues nsim=1/1000 {

*print i

di "`nsim'"

foreach b_yx of numlist 0 0.5 1 {

foreach b_ry of numlist 0 0.5 1 {

foreach b_rz of numlist 0 0.5 1 {

clear

quietly set obs 1000

gen z=rnormal(0,1)

gen x=rnormal(0,1)

gen y=rbinomial(1,invlogit(`b_yx'*x ))

gen r=rnormal(`b_ry'*y + `b_rz'*z,1)

gen ymiss=y

quietly replace ymiss=. if r>0

quietly save `tmpfull', replace

*MI with X

quietly mi set flong

quietly mi register imputed ymiss

quietly mi register regular x

quietly mi impute logit ymiss = x, add(5)

quietly mi estimate: logistic ymiss x

local beta_x = e(b_mi)[1,1]

local se_x = sqrt(e(V_mi)[1,1])

*MI with X and Z

*restore simulated data i.e. before imputation

use `tmpfull', clear

quietly mi set flong

quietly mi register imputed ymiss

quietly mi register regular x z

quietly mi impute logit ymiss = x z, add(5)

quietly mi estimate: logistic ymiss x

post `simloop' (`nsim') (`b_yx') (`b_ry') (`b_rz') (`beta_x') (`se_x') (e(b_mi)[1,1]) (sqrt(e(V_mi)[1,1]))

}

}

}

}

postclose `simloop'

*** Scenario 2 ***

* 1. Verification of bias

*Define postfile to store results

tempname simloop

postfile `simloop' int(i) float(b_yx beta_yx beta_yx_cond_r beta_yx_cond_rz beta_xy_cond_r beta_xy_cond_rz theor_beta_xy_cond_r theor_beta_xy_cond_rz) using "sim_yxzru_scen2.dta", replace

forvalues i=1/1000 {

*di "`i'"

clear

*SD

local s_z=runiform(0,2)

local s_r=runiform(0,2)

local s_x=runiform(0,2)

local s_y=runiform(0,2)

local s_u=runiform(0,2)

*beta

local b_yx=runiform(0,2)

local b_yu=runiform(0,2)

local b_rz=runiform(0,2)

local b_ru=runiform(0,2)

*RVs

quietly set obs 100000

gen z=rnormal(0,`s_z')

gen x=rnormal(0,`s_x')

gen u=rnormal(0,`s_u')

gen y=rnormal(`b_yx'*x + `b_yu'*u,`s_y')

gen r=rnormal(`b_ru'*u + `b_rz'*z,`s_r')

*Estimate beta_YX

quietly regress y x

local beta_yx=e(b)[1,1]

*Estimate beta_YX|R

quietly regress y x r

*Store estimate

local beta_yx_cond_r=e(b)[1,1]

*Estimate beta_YX|R,Z

quietly regress y x r z

*Store estimate

local beta_yx_cond_rz=e(b)[1,1]

*Estimate beta_XY|R

quietly regress x y r

*Store estimate

local beta_xy_cond_r=e(b)[1,1]

*Estimate beta_XY|R,Z

quietly regress x y r z

*Store estimate

local beta_xy_cond_rz=e(b)[1,1]

*Calculate theoretical values

local theor_beta_xy_cond_r =(`b_yx'*`s_x'^2/(`b_yx'^2*`s_x'^2 + `b_yu'^2*`s_u'^2 + `s_y'^2)) * 1/(1-(`b_yu'^2*`b_ru'^2*`s_u'^4/((`b_yx'^2*`s_x'^2 + `b_yu'^2*`s_u'^2 + `s_y'^2)*(`b_rz'^2*`s_z'^2 + `b_ru'^2*`s_u'^2 + `s_r'^2))))

local theor_beta_xy_cond_rz =(`b_yx'*`s_x'^2/(`b_yx'^2*`s_x'^2 + `b_yu'^2*`s_u'^2 + `s_y'^2)) * 1/(1-(`b_yu'^2*`b_ru'^2*`s_u'^4/((`b_yx'^2*`s_x'^2 + `b_yu'^2*`s_u'^2 + `s_y'^2)*(`b_ru'^2*`s_u'^2 + `s_r'^2))))

post `simloop' (`i') (`b_yx') (`beta_yx') (`beta_yx_cond_r') (`beta_yx_cond_rz') (`beta_xy_cond_r') (`beta_xy_cond_rz') (`theor_beta_xy_cond_r') (`theor_beta_xy_cond_rz')

}

postclose `simloop'

use sim_yxzru_scen2, clear

gen bias_beta_yx_cond_r=beta_yx_cond_r-beta_yx

gen bias_beta_yx_cond_rz=beta_yx_cond_rz-beta_yx

gen diff_beta_xy_cond_r=theor_beta_xy_cond_r-beta_xy_cond_r

gen diff_beta_xy_cond_rz=theor_beta_xy_cond_rz-beta_xy_cond_rz

sum bias_beta_yx_cond_r bias_beta_yx_cond_rz diff_beta_xy_cond_r diff_beta_xy_cond_rz, detail

* 2. Additional bias – X continuous

****** Varying the size of coefficients - still use mu_z=0 and mu_u=0 and all error vars = 1*******

tempname simloop

postfile `simloop' int(nsim) float(b_yx b_yu b_ru b_rz beta_x_y se_x_y beta_x_yz se_x_yz) using "MIaddbias_illustration_scen2Xcts.dta", replace

*Create a temporary file for storing simulated data

tempfile tmpfull

forvalues nsim=1/1000 {

*print i

di "`nsim'"

foreach b_yx of numlist 0 0.5 1 {

foreach b_yu of numlist 0 0.5 1 {

foreach b_ru of numlist 0 0.5 1 {

foreach b_rz of numlist 0 0.5 1 {

clear

quietly set obs 1000

gen z=rnormal(0,1)

gen x=rnormal(0,1)

gen u=rnormal(0,1)

gen y=rnormal(`b_yx'*x + `b_yu'*u,1)

gen r=rnormal(`b_ru'*u + `b_rz'*z,1)

gen xmiss=x

quietly replace xmiss=. if r>0

quietly save `tmpfull', replace

*MI with Y

quietly mi set flong

quietly mi register imputed xmiss

quietly mi register regular y

quietly mi impute regress xmiss = y, add(5)

quietly mi estimate: regress y xmiss

local beta_x = e(b_mi)[1,1]

local se_x = sqrt(e(V_mi)[1,1])

*MI with X and Z

*restore simulated data i.e. before imputation

use `tmpfull', clear

quietly mi set flong

quietly mi register imputed xmiss

quietly mi register regular y z

quietly mi impute regress xmiss = y z, add(5)

quietly mi estimate: regress y xmiss

post `simloop' (`nsim') (`b_yx') (`b_yu') (`b_ru') (`b_rz') (`beta_x') (`se_x') (e(b_mi)[1,1]) (sqrt(e(V_mi)[1,1]))

}

}

}

}

}

postclose `simloop'

* 3. Additional bias - Binary X

tempname simloop

postfile `simloop' int(nsim) float(b_yx b_yu b_ru b_rz beta_x_y se_x_y beta_x_yz se_x_yz) using "MIaddbias_illustration_scen2Xbin.dta", replace

*Create a temporary file for storing simulated data

tempfile tmpfull

forvalues nsim=1/1000 {

di "`nsim'"

foreach b_yx of numlist 0 0.5 1 {

foreach b_yu of numlist 0 0.5 1 {

foreach b_ru of numlist 0 0.5 1 {

foreach b_rz of numlist 0 0.5 1 {

clear

quietly set obs 1000

gen z=rnormal(0,1)

gen x=rbinomial(1,0.5)

gen u=rnormal(0,1)

gen y=rnormal(`b_yx'*x + `b_yu'*u,1)

gen r=rnormal(`b_ru'*u + `b_rz'*z,1)

gen xmiss=x

quietly replace xmiss=. if r>0

quietly save `tmpfull', replace

*MI with Y

quietly mi set flong

quietly mi register imputed xmiss

quietly mi register regular y

quietly mi impute logit xmiss = y, add(5)

quietly mi estimate: regress y xmiss

local beta_x = e(b_mi)[1,1]

local se_x = sqrt(e(V_mi)[1,1])

*MI with X and Z

*restore simulated data i.e. before imputation

use `tmpfull', clear

quietly mi set flong

quietly mi register imputed xmiss

quietly mi register regular y z

quietly mi impute logit xmiss = y z, add(5)

quietly mi estimate: regress y xmiss

post `simloop' (`nsim') (`b_yx') (`b_yu') (`b_ru') (`b_rz') (`beta_x') (`se_x') (e(b_mi)[1,1]) (sqrt(e(V_mi)[1,1]))

}

}

}

}

}

postclose `simloop'

* 4. Additional bias - Binary Y

tempname simloop

postfile `simloop' int(nsim) float(b_yx b_yu b_ru b_rz beta_x_marg se_x_marg beta_x_cra se_x_cra beta_x_miy se_x_miy beta_x_miyz se_x_miyz) using "MIaddbias_illustration_scen2Ybin.dta", replace

*Create a temporary file for storing simulated data

tempfile tmpfull

forvalues nsim=1/1000 {

di "`nsim'"

foreach b_yx of numlist 0 0.5 1 {

foreach b_yu of numlist 0 0.5 1 {

foreach b_ru of numlist 0 0.5 1 {

foreach b_rz of numlist 0 0.5 1 {

clear

quietly set obs 1000

gen z=rnormal(0,1)

gen x=rnormal(0,1)

gen u=rnormal(0,1)

gen y=rbinomial(1,invlogit(`b_yx'*x + `b_yu'*u))

gen r=rnormal(`b_ru'*u + `b_rz'*z,1)

gen ymiss=y

quietly replace ymiss=. if r>0

quietly save `tmpfull', replace

*Full data estimate

quietly logit y x

local beta_xmarg=e(b)[1,1]

local se_xmarg=sqrt(e(V)[1,1])

*CRA

quietly logit ymiss x

local beta_xcra=e(b)[1,1]

local se_xcra=sqrt(e(V)[1,1])

*MI with Y

quietly mi set flong

quietly mi register imputed ymiss

quietly mi register regular x

quietly mi impute logit ymiss = x, add(5)

quietly mi estimate: logit ymiss x

local beta_x = e(b_mi)[1,1]

local se_x = sqrt(e(V_mi)[1,1])

*MI with X and Z

*restore simulated data i.e. before imputation

use `tmpfull', clear

quietly mi set flong

quietly mi register imputed ymiss

quietly mi register regular x z

quietly mi impute logit ymiss = x z, add(5)

quietly mi estimate: logit ymiss x

post `simloop' (`nsim') (`b_yx') (`b_yu') (`b_ru') (`b_rz') (`beta_xmarg') (`se_xmarg') (`beta_xcra') (`se_xcra') (`beta_x') (`se_x') (e(b_mi)[1,1]) (sqrt(e(V_mi)[1,1]))

}

}

}

}

}

postclose `simloop'

*** Scenario 3 ***

*** Setting 1: Y partially observed ***

* 1. Verification of bias

*Define postfile to store results

tempname simloop

postfile `simloop' int(i) float(b_yx beta_yx beta_yx_cond_r beta_yx_cond_rz theor_bias emp_bias theor_addbias emp_addbias) using "sim_yxzr_scen3.dta", replace

forvalues i=1/1000 {

*di "`i'"

clear

*SD

local s_z=runiform(0,2)

local s_r=runiform(0,2)

local s_x=runiform(0,2)

local s_y=runiform(0,2)

*beta

local b_yx=runiform(0,2)

local b_ry=runiform(0,2)

local b_rx=runiform(0,2)

local b_rz=runiform(0,2)

*RVs

quietly set obs 100000

gen z=rnormal(0,`s_z')

gen x=rnormal(0,`s_x')

gen y=rnormal(`b_yx'*x,`s_y')

gen r=rnormal(`b_ry'*y + `b_rx'*x + `b_rz'*z,`s_r')

*Estimate beta_YX

quietly regress y x

local beta_yx=e(b)[1,1]

*Estimate beta_YX|R

quietly regress y x r

*Store estimate

local beta_yx_cond_r=e(b)[1,1]

*Estimate beta_YX|R,Z

quietly regress y x r z

*Store estimate

local beta_yx_cond_rz=e(b)[1,1]

*Calculate theoretical and empirical bias and bias amp

local emp_bias = `beta_yx_cond_r' - `beta_yx'

local emp_addbias = `beta_yx_cond_rz' - `beta_yx_cond_r'

local theor_bias=-`b_yx'*`b_ry'*`s_y'^2*(`b_ry' + (`b_rx'/`b_yx'))/(`b_ry'^2*`s_y'^2 + `s_r'^2 + `b_rz'^2*`s_z'^2)

local theor_addbias=`b_yx'*`b_ry'*`s_y'^2*(`b_ry' + (`b_rx'/`b_yx'))*((1/(`b_ry'^2*`s_y'^2 + `s_r'^2 + `b_rz'^2*`s_z'^2)) - (1/(`b_ry'^2*`s_y'^2 + `s_r'^2)))

post `simloop' (`i') (`b_yx') (`beta_yx') (`beta_yx_cond_r') (`beta_yx_cond_rz') (`theor_bias') (`emp_bias') (`theor_addbias') (`emp_addbias')

}

postclose `simloop'

* 2. Illustration of max additional bias equation

*Define postfile to store results

postfile `simloop' float(b_yx b_ry b_rz b_rx max_addbias) using "maxaddbias_illustration_scen3Ycts.dta", replace

foreach b_yx of numlist 0 0.25 0.5 0.75 1 {

foreach b_ry of numlist 0 0.25 0.5 0.75 1 {

foreach b_rz of numlist 0 0.25 0.5 0.75 1 {

foreach b_rx of numlist 0 0.25 0.5 0.75 1 {

clear

*Write in a different form to avoid division by zero

local max_addbias=(`b_yx'*`b_ry'^2 + `b_ry'*`b_rx')*((1/(`b_ry'^2 + 1 + `b_rz'^2)) - (1/(`b_ry'^2 + 1)))

post `simloop' (`b_yx') (`b_ry') (`b_rz') (`b_rx') (`max_addbias')

}

}

}

}

postclose `simloop'

* 3. Binary Y

tempname simloop

postfile `simloop' int(nsim) float(b_yx b_ry b_rz b_rx beta_x_x se_x_x beta_x_xz se_x_xz) using "MIaddbias_illustration_scen3Ybin.dta", replace

*Create a temporary file for storing simulated data

tempfile tmpfull

forvalues nsim=1/1000 {

di "`nsim'"

foreach b_yx of numlist 0 0.5 1 {

foreach b_ry of numlist 0 0.5 1 {

foreach b_rz of numlist 0 0.5 1 {

foreach b_rx of numlist 0 0.5 1 {

clear

quietly set obs 1000

gen z=rnormal(0,1)

gen x=rnormal(0,1)

gen y=rbinomial(1,invlogit(`b_yx'*x ))

gen r=rnormal(`b_ry'*y + `b_rx'*x + `b_rz'*z,1)

gen ymiss=y

quietly replace ymiss=. if r>0

quietly save `tmpfull', replace

*MI with X

quietly mi set flong

quietly mi register imputed ymiss

quietly mi register regular x

quietly mi impute logit ymiss = x, add(5)

quietly mi estimate: logistic ymiss x

local beta_x = e(b_mi)[1,1]

local se_x = sqrt(e(V_mi)[1,1])

*MI with X and Z

*restore simulated data i.e. before imputation

use `tmpfull', clear

quietly mi set flong

quietly mi register imputed ymiss

quietly mi register regular x z

quietly mi impute logit ymiss = x z, add(5)

quietly mi estimate: logistic ymiss x

post `simloop' (`nsim') (`b_yx') (`b_ry') (`b_rz') (`b_rx') (`beta_x') (`se_x') (e(b_mi)[1,1]) (sqrt(e(V_mi)[1,1]))

}

}

}

}

}

postclose `simloop'

*** Setting 2. X partially observed ***

* 1. Verification of maximum bias of the Y coefficient

*Define postfile to store results

tempname simloop

postfile `simloop' int(i) float(b_yx beta_xy beta_xy_cond_r beta_xy_cond_rz theor_bias emp_bias theor_addbias emp_addbias) using "sim_yxzr_scen3_impX.dta", replace

forvalues i=1/1000 {

clear

*SD

local s_z=runiform(0,2)

local s_r=runiform(0,2)

local s_x=runiform(0,2)

local s_y=runiform(0,2)

*beta

local b_yx=runiform(0,2)

local b_ry=runiform(0,2)

local b_rx=runiform(0,2)

local b_rz=runiform(0,2)

*RVs

quietly set obs 100000

gen z=rnormal(0,`s_z')

gen x=rnormal(0,`s_x')

gen y=rnormal(`b_yx'*x,`s_y')

gen r=rnormal(`b_ry'*y + `b_rx'*x + `b_rz'*z,`s_r')

*Estimate beta_XY

quietly regress x y

local beta_xy=e(b)[1,1]

*Estimate beta_XY|R

quietly regress x y r

*Store estimate

local beta_xy_cond_r=e(b)[1,1]

*Estimate beta_XY|R,Z

quietly regress x y r z

*Store estimate

local beta_xy_cond_rz=e(b)[1,1]

*Calculate theoretical and empirical bias and bias amp

local emp_bias = `beta_xy_cond_r' - `beta_xy'

local emp_addbias = `beta_xy_cond_rz' - `beta_xy_cond_r'

local b_xy=`b_yx'*`s_x'^2/(`b_yx'^2*`s_x'^2 + `s_y'^2)

local theor_bias=-`b_xy'*`b_rx'*(`b_ry'*`s_y'^2/`b_yx' + `b_rx'*`s_x'^2*(1 - `b_yx'^2*`s_x'^2/(`b_yx'^2*`s_x'^2+`s_y'^2)))/(`b_rx'^2*`s_x'^2 + `s_r'^2 + `b_rz'^2*`s_z'^2 - (`b_yx'^2*`b_rx'^2*`s_x'^4/(`b_yx'^2*`s_x'^2+`s_y'^2)))

local theor_addbias=`b_xy'*`b_rx'*((`b_ry'*`s_y'^2/`b_yx') + `b_rx'*`s_x'^2*(1 - (`b_yx'^2*`s_x'^2/(`b_yx'^2*`s_x'^2+`s_y'^2))))* ///

((1/(`b_rx'^2*`s_x'^2 + `s_r'^2 + `b_rz'^2*`s_z'^2 - (`b_yx'^2*`b_rx'^2*`s_x'^4/(`b_yx'^2*`s_x'^2+`s_y'^2)))) - (1/(`b_rx'^2*`s_x'^2 + `s_r'^2 - (`b_yx'^2*`b_rx'^2*`s_x'^4/(`b_yx'^2*`s_x'^2+`s_y'^2)))))

post `simloop' (`i') (`b_xy') (`beta_xy') (`beta_xy_cond_r') (`beta_xy_cond_rz') (`theor_bias') (`emp_bias') (`theor_addbias') (`emp_addbias')

}

postclose `simloop'

* 2. Bias illustration

****** Varying the size of coefficients - still use mu_z=0 and mu_u=0 and all error vars = 1*******

tempname simloop

postfile `simloop' int(nsim) float(b_yx b_ry b_rz b_rx beta_x_x se_x_x beta_x_xz se_x_xz) using "MIaddbias_illustration_scen3Xcts.dta", replace

*Create a temporary file for storing simulated data

tempfile tmpfull

forvalues nsim=1/1000 {

*print i

di "`nsim'"

foreach b_yx of numlist 0 0.5 1 {

foreach b_ry of numlist 0 0.5 1 {

foreach b_rz of numlist 0 0.5 1 {

foreach b_rx of numlist 0 0.5 1 {

clear

quietly set obs 1000

gen z=rnormal(0,1)

gen x=rnormal(0,1)

gen y=rnormal(`b_yx'*x,1)

gen r=rnormal(`b_ry'*y + `b_rx'*x + `b_rz'*z,1)

gen xmiss=x

quietly replace xmiss=. if r>0

quietly save `tmpfull', replace

*MI with Y

quietly mi set flong

quietly mi register imputed xmiss

quietly mi register regular y

quietly mi impute regress xmiss = y, add(5)

quietly mi estimate: regress y xmiss

local beta_x = e(b_mi)[1,1]

local se_x = sqrt(e(V_mi)[1,1])

*MI with Y and Z

*restore simulated data i.e. before imputation

use `tmpfull', clear

quietly mi set flong

quietly mi register imputed xmiss

quietly mi register regular y z

quietly mi impute regress xmiss = y z, add(5)

quietly mi estimate: regress y xmiss

post `simloop' (`nsim') (`b_yx') (`b_ry') (`b_rz') (`b_rx') (`beta_x') (`se_x') (e(b_mi)[1,1]) (sqrt(e(V_mi)[1,1]))

}

}

}

}

}

postclose `simloop'

* 3. Additional bias - Binary X

****** Varying the size of coefficients - still use mu_z=0 and mu_u=0 and all error vars = 1*******

tempname simloop

postfile `simloop' int(nsim) float(b_yx b_ry b_rz b_rx beta_x_x se_x_x beta_x_xz se_x_xz) using "MIaddbias_illustration_scen3Xbin.dta", replace

*Create a temporary file for storing simulated data

tempfile tmpfull

forvalues nsim=1/1000 {

*print i

di "`nsim'"

foreach b_yx of numlist 0 0.5 1 {

foreach b_ry of numlist 0 0.5 1 {

foreach b_rz of numlist 0 0.5 1 {

foreach b_rx of numlist 0 0.5 1 {

clear

quietly set obs 1000

gen z=rnormal(0,1)

gen x=rbinomial(1,0.5)

gen y=rnormal(`b_yx'*x,1)

gen r=rnormal(`b_ry'*y + `b_rx'*x + `b_rz'*z,1)

gen xmiss=x

quietly replace xmiss=. if r>0

quietly save `tmpfull', replace

*MI with Y

quietly mi set flong

quietly mi register imputed xmiss

quietly mi register regular y

quietly mi impute logit xmiss = y, add(5)

quietly mi estimate: regress y xmiss

local beta_x = e(b_mi)[1,1]

local se_x = sqrt(e(V_mi)[1,1])

*MI with Y and Z

*restore simulated data i.e. before imputation

use `tmpfull', clear

quietly mi set flong

quietly mi register imputed xmiss

quietly mi register regular y z

quietly mi impute logit xmiss = y z, add(5)

quietly mi estimate: regress y xmiss

post `simloop' (`nsim') (`b_yx') (`b_ry') (`b_rz') (`b_rx') (`beta_x') (`se_x') (e(b_mi)[1,1]) (sqrt(e(V_mi)[1,1]))

}

}

}

}

}

postclose `simloop'

*Section S7. Stata code to perform the real data analysis*

* 1. Check mDAG associations

*IQ15 and maternal smoking

regress iq15 i.matsmoki i.bf_bin i.sex i.msoc_prof_nonman i.mated_16plus i.parity_cat matage i.housing_cat

*Predictors of R_IQ15

logistic comp_caseiq15 i.matsmoki i.bf_bin i.sex i.msoc_prof_nonman i.mated_16plus i.parity_cat matage i.housing_cat, coef

*2. CRA

regress iq15 i.bf_bin i.sex i.msoc_prof_nonman i.mated_16plus i.parity_cat matage i.housing_cat

*3. MI using i.bfduration i.mated i.msoc to predict iq15 and vice versa

quietly mi set flong

quietly mi register imputed iq15 bf_bin msoc_prof_nonman mated_16plus parity_cat housing_cat

quietly mi register regular sex matage

mi impute chained (regress) iq15 (logit) bf_bin (ologit) parity_cat (mlogit) housing_cat (logit) msoc_prof_nonman mated_16plus = i.sex matage, ///

add(100) burnin(20) /*dryrun*/ dots

mi estimate: regress iq15 i.bf_bin i.sex i.msoc_prof_nonman i.mated_16plus i.parity_cat matage i.housing_cat

* 4. MI additionally using matsmok

use alspac_subv1, clear

count

*13923

quietly mi set flong

quietly mi register imputed iq15 bf_bin msoc_prof_nonman mated_16plus parity_cat housing_cat matsmoki

quietly mi register regular sex matage

mi impute chained (regress) iq15 (logit) bf_bin (ologit) parity_cat (mlogit) housing_cat (logit) msoc_prof_nonman mated_16plus matsmoki = i.sex matage, ///

add(100) burnin(20) /*dryrun*/ dots

mi estimate: regress iq15 i.bf_bin i.sex i.msoc_prof_nonman i.mated_16plus i.parity_cat matage i.housing_cat

* 5. Map to theor bias

corr bf_bin matsmoki, covariance

gen var_X=r(Var_1)

gen var_Z=r(Var_2)

logistic comp_caseiq15 i.matsmoki i.bf_bin i.sex i.msoc_prof_nonman i.mated_16plus i.parity_cat matage i.housing_cat, coef

gen logOR_Z=e(b)[1,2]

gen logOR_X=e(b)[1,4]

/* Substituting in all terms gives: */

gen maxbiasamp=1+((0.6*logOR_Z)^2*var_Z/(1-(0.6*logOR_Z)^2*var_Z-(0.6*logOR_X)^2*var_X))
